# How to design optimal brain stimulation to modulate phase-amplitude coupling?

**DOI:** 10.1088/1741-2552/ad5b1a

**Published:** 2024-07-10

**Authors:** Benoit Duchet, Rafal Bogacz

**Affiliations:** MRC Brain Network Dynamics Unit, Nuffield Department of Clinical Neuroscience, University of Oxford, Oxford, United Kingdom

**Keywords:** phase-amplitude coupling, brain stimulation, waveform optimisation, phase-locked stimulation, neurological disorders

## Abstract

**Objective:**

Phase-amplitude coupling (PAC), the coupling of the amplitude of a faster brain rhythm to the phase of a slower brain rhythm, plays a significant role in brain activity and has been implicated in various neurological disorders. For example, in Parkinson’s disease, PAC between the beta (13−30 Hz) and gamma (30−100 Hz) rhythms in the motor cortex is exaggerated, while in Alzheimer’s disease, PAC between the theta (4−8 Hz) and gamma rhythms is diminished. Modulating PAC (i.e. reducing or enhancing PAC) using brain stimulation could therefore open new therapeutic avenues. However, while it has been previously reported that phase-locked stimulation can increase PAC, it is unclear what the optimal stimulation strategy to modulate PAC might be. Here, we provide a theoretical framework to narrow down the experimental optimisation of stimulation aimed at modulating PAC, which would otherwise rely on trial and error.

**Approach:**

We make analytical predictions using a Stuart−Landau model, and confirm these predictions in a more realistic model of coupled neural populations.

**Main results:**

Our framework specifies the critical Fourier coefficients of the stimulation waveform which should be tuned to optimally modulate PAC. Depending on the characteristics of the amplitude response curve of the fast population, these components may include the slow frequency, the fast frequency, combinations of these, as well as their harmonics. We also show that the optimal balance of energy between these Fourier components depends on the relative strength of the endogenous slow and fast rhythms, and that the alignment of fast components with the fast rhythm should change throughout the slow cycle. Furthermore, we identify the conditions requiring to phase-lock stimulation to the fast and/or slow rhythms.

**Significance:**

Together, our theoretical framework lays the foundation for guiding the development of innovative and more effective brain stimulation aimed at modulating PAC for therapeutic benefit.

## 1 Introduction

Phase-amplitude coupling (PAC), a type of cross-frequency coupling where the amplitude of faster brain oscillations is coupled to the phase of slower brain oscillations, is widespread across species and brain regions. Most notably, PAC was shown to be implicated in memory and learning, in particular through coupling of the amplitude of the gamma rhythm (30−100 Hz) to the phase of the theta rhythm (4−8 Hz) in the hippocampus [1–6]. Beyond memory processes, PAC is for example modulated during movement and speech [7], visual attention [8], auditory processing [9], complex cognitive function [10], as well as during development [11].

PAC was also found to be abnormal in various neurological disorders—see [12] for a review. In Parkinson’s disease (PD), coupling between the beta phase (13−30 Hz) and gamma amplitude in the motor cortex is exaggerated compared to patients with dystonia and patients with epilepsy, both at rest and during movement [13]. Elevated PAC was reported in patients with PD off dopaminergic medication compared to patients on medication, as well as compared to humans without a movement disorder [14]. This increased PAC is reduced by deep brain stimulation (DBS) [15]. Similarly, alpha (8−12 Hz) gamma PAC is exaggerated in the sensorimotor cortex of patients with essential tremor [16]. As expected from its involvement in memory, theta-gamma PAC is impacted in Alzheimer’s disease (AD). Lower theta-gamma PAC than controls was found in AD rodent models [17, 18], with alterations appearing before significant accumulation of amyloid beta in some animals [19]. In humans, theta-gamma PAC was lower in patients with mild cognitive impairment compared to healthy age-matched participants, lower still in patients with AD [20], and correlated with cognitive and memory performance [20, 21]. PAC was also reported to be elevated during epileptic seizures [22]. Furthermore, PAC was suggested as a biomarker for brain-computer interface-mediated motor recovery in chronic stroke [23], and for rehabilitation of speech discrimination in cochlear implant users [24].

Given changes in PAC from healthy levels in neurological disorders, in some cases correlated with symptoms or recovery, restoring healthy PAC levels is a promising target for neuromodulation therapies. However, how to stimulate to enhance or decrease PAC levels has received very little attention to date. A notable exception is the work by Salimpour and colleagues, which showed that phase-locking motor cortical electrical stimulation to the peak of the beta rhythm increased beta-gamma PAC in humans compared to baseline, and compared to stimulation phase-locked to the trough of the beta rhythm [25]. Similarly, phase-locking hippocampal transcranial ultrasound stimulation to the peak of the theta rhythm increased theta-gamma PAC in rats [26]. Nevertheless, it is unclear what the optimal stimulation strategy to enhance or decrease PAC might be. Here, we develop a theoretical framework to address this question using the analytically tractable Stuart−Landau (SL) model as well as a more biologically realistic neural mass model, the Wilson−Cowan (WC) model. While we focus on PAC-enhancing stimulation (which could be of interest for example in patients with AD), the same framework can be applied to stimulation aimed at reducing PAC. Our framework is directly applicable to neuromodulation modalities where the Fourier coefficients of the stimulation waveform can be tuned, such as transcranial alternating current stimulation (tACS). For modalities that can only generate square pulses (e.g. DBS), the optimal waveforms predicted from our theoretical framework can be approximated by pulsatile waveforms.

## 2 Results

We develop a theory of optimal PAC-enhancing stimulation using the SL model, which offers the possibility of analytical insights. In particular, we build on two key mechanisms contributing to increasing PAC, namely stimulation at the slow frequency, and stimulation with a modulated component at the fast frequency. We show that whether these mechanisms can be leveraged depends on characteristics of the fast population’s response to stimulation. We proceed to verify elements of the theory in a neural mass model, the WC model. We finish by considering practical questions, in particular the balance of the Fourier coefficients of the stimulation waveform as a function of the strength of the endogenous slow and fast rhythms, the necessity (or lack thereof) of phase-locking stimulation to the fast and/or slow rhythm, and how to approximate the optimal waveforms with pulsatile waveforms. Flowcharts that could guide experimentalists in designing optimal-PAC modulating stimulation are presented in figure 10.

### 2.1 Developing optimal PAC-enhancing stimulation in the Stuart−Landau model

The SL model is arguably the simplest phase-amplitude model used in neuroscience [27–30], and is therefore ideally suited to obtain analytical insights on PAC-enhancing stimulation. The model represents the canonical form of a Hopf bifurcation, and can therefore operate in the fixed-point or limit-cycle regime. We are considering a SL population operating at the fast frequency of interest with order parameter $z_{f}=\rho_{f}{\text{e}}^{\text{i}\theta_{f}}$ (oscillation amplitude *ρ_f_* and oscillation phase *θ_f_*) evolving according to

$${\dot{z}}_{f}=\left(\delta +{\text{i}\omega }_{f}-{\left|z_{f}\right|}^{2}\right)z_{f},$$

where *ω_f_* = 2*πf_f_* is the angular frequency of the fast population, and *δ* a bifurcation parameter. When *δ* > 0, the fast population is in the limit-cycle regime and generates intrinsic oscillations of amplitude converging to $\sqrt{\delta }$. When *δ* ⩽ 0, the fast population is in a quiescent state (fixed-point regime).

**Figure 1:**
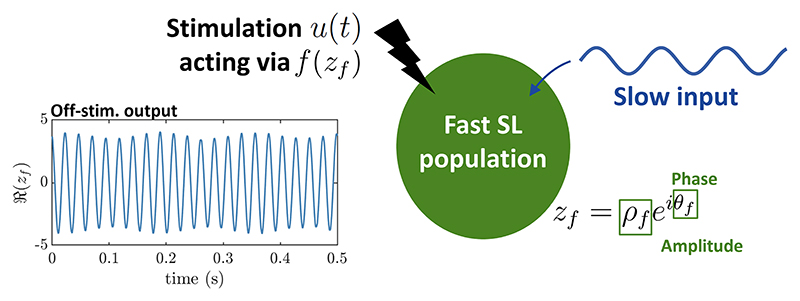
Sketch of the Stuart−Landau model with stimulation. Intrinsic PAC is generated by a slow input (shown in dark blue) interacting with a fast Stuart−Landau population (represented in green). An example of the output of the fast population (real part of the fast-population order parameter) displaying PAC in the absence of stimulation is shown in the left panel. The stimulation *u*(*t*) (in black) acts on the fast population via a stimulation coupling function *f*(*z_f_*), where *z_f_* is the order parameter of the fast population with oscillation amplitude *ρ_f_* and oscillation phase *θ*_f_.

We assume that neither stimulation nor the fast rhythm significantly affects the slow rhythm contributing to PAC, and we model the slow rhythm as an input to the fast population. In the case of hippocampal theta-gamma PAC, the slow input can represent the theta input from pacemaker neurons in the medial septum for example, believed to be the main contributor to hippocampal theta [31–33]. We present the limitations of this approach in the Discussion. We further assume that the slow input of strength *k_s_* and angular frequency *ω_s_* = 2*πf_s_* is coupled to the fast population through its mean-field (see figure 1), thereby affecting the amplitude (but not the phase) of the fast oscillations. As shown by Quin and colleagues, such a slow input can generate PAC [27] in the SL model. Indeed, since

$${\dot{z}}_{f}=\left(\delta +{\text{i}\omega }_{f}-{\left|z_{f}\right|}^{2}\right)z_{f}+z_{f}k_{s}\text{cos}\omega_{s}t$$

can be re-written as

$${\dot{z}}_{f}=\left(\delta +k_{s}\text{cos}\omega_{s}t+\text{i}\omega_{f}-{\left|z_{f}\right|}^{2}\right)z_{f},$$

the parameter controlling the amplitude of the fast oscillations becomes *δ*_PAC_ = *δ* + *k_s_* cos *ω*_*s*_*t*. This means that the amplitude of the fast oscillations is controlled by the phase of the slow input.

In the next sections, we will optimise the stimulation waveform to maximally increase PAC for a given stimulation energy budget. We will consider a stimulation input *u*(*t*) provided to the fast SL population receiving a slow input. The stimulation is provided to the population through the stimulation coupling function *f* (its connection with experimental measures is detailed below). The evolution of the order parameter of the fast SL population is given by

(1) $${\dot{z}}_{f}=\left(\delta +k_{s}\text{cos}\omega_{s}t+\text{i}\omega_{f}-{\left|z_{f}\right|}^{2}\right)z_{f}+f\left(z_{f}\right)u(t).$$

In what follows, we expand *u*(*t*) as a truncated Fourier Series

(2) $$u(t)=\overset{N_{u}}{\underset{\begin{array}{c} n=-N_{u}\\ n\neq 0 \end{array}}{\sum }}u_{n}e^{n\text{i}\omega_{s}t}=\overset{N_{u}}{\underset{n=1}{\sum }}\left[a_{n}\text{cos}\left(n\omega_{s}t\right)+b_{n}\text{sin}\left(n\omega_{s}t\right)\right],$$

with complex coefficients *u_n_*, or equivalently real coefficients *a_n_* and *b_n_*, and with truncation order *N_u_*. To enforce charge balance of the stimulus (a requirement for brain stimulation to avoid tissue damage), the zeroth-order coefficient is zero.

We will show in the next sections that the way stimulation is coupled to the fast population, and in particular the amplitude response curve (ARC) of the fast population, determines which Fourier coefficients contribute to modulating PAC. The stimulation coupling function is directly related to the ARC and the phase response curve (PRC) of the fast population. Here, the ARC describes the instantaneous change in amplitude of the collective activity of a neural population (e.g. measured in the local field potential [34, 35]) due to stimulation. The ARC is a function of the state of the neural population, e.g. the phase and/or amplitude of the collective oscillation when stimulation is received. Similarly, we take the PRC to refer to the instantaneous change in phase of the collective oscillation due to stimulation, as a function of the state of the neural population. Note that in some studies, the PRC refers instead to the response of individual neurons (e.g. [36–38]). This is in contrast with this study, where we consider changes on the population level. Given these definitions, we have $\text{ARC}(z)u(t)=\dot{\rho }$stim and $\text{PRC}(z)u(t)={\dot{\theta }}_{\text{stim}}$, where ${\dot{\rho }}_{\text{stim}}$ and ${\dot{\theta }}_{\text{stim}}$ are the instantaneous changes in amplitude and phase of the neural population due to stimulation, respectively, and *z* is the order parameter of the SL model. Using the product rule on the definition of the order parameter, we have $\dot{z}=\dot{\rho }{\text{e}}^{\text{i}\theta }+\text{i}\dot{\theta }\rho {\text{e}}^{\text{i}\theta }$. Without loss of gen-erality, the instantaneous change in *z* due to stimulation at time *t* can therefore be written as ${\dot{z}}_{\text{stim}}=\left[\text{ARC}(z){\text{e}}^{\text{i}\theta }+\text{iPRC}(z)z\right]u(t)$. Since ${\dot{z}}_{\text{stim}}=f\left(z_{f}\right)u(t)$, we can identify the stimulation coupling function as

(3) $$f\left(z_{f}\right)=\text{ARC}\left(z_{f}\right){\text{e}}^{\text{i}\theta_{f}}+\text{iPRC}\left(z_{f}\right)z_{f}.$$

**Figure 2:**
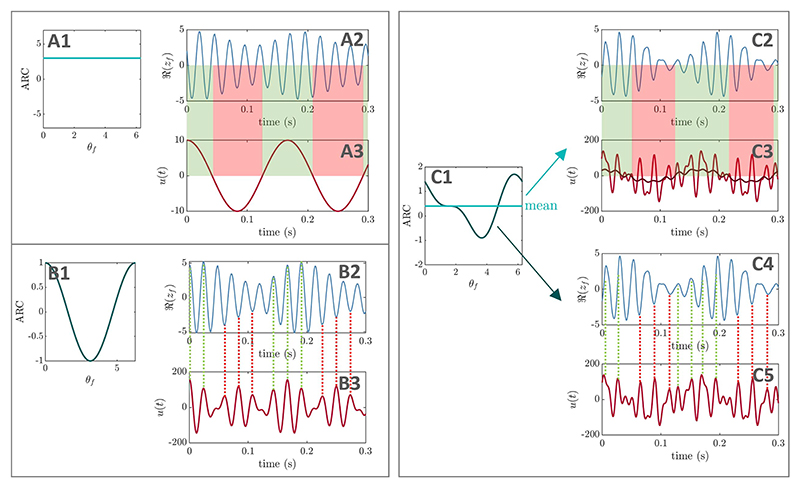
PAC-enhancing mechanisms in the Stuart−Landau model depend on the amplitude response of the fast population. Panel (A) corresponds to foundational case one, where the amplitude response of the fast population does not depend on its phase ((A1), shown for *ρ_f_* = 3). Sinusoidal stimulation (A3) therefore enhances the amplitude of the fast population (A2) when the stimulation waveform is positive (green highlights), and suppresses the amplitude of the fast population when it is negative (red highlights). Panel (B) corresponds to foundational case two, where the amplitude response of the fast population depends on its phase but has zero mean (B1). Where the fast-oscillation amplitude (B2) should be increased, the optimal stimulation waveform ((B3), taken from figure 4(C)) has fast-frequency components aligned with the peak of the fast rhythm (green dashes). Conversely, where the fast-oscillation amplitude should be decreased, the optimal stimulation waveform has fast-frequency components anti-phase-aligned with the peak of the fast rhythm. Panel (C) corresponds to a general case where the amplitude response of the fast population does depend on its phase and has a non-zero mean (highlighted in light green in (C1)). The optimal stimulation waveform (taken from figure 5(C)) combines mechanisms of PAC-enhancement from panels (A) and (B), as shown in panels (C2)−(C5). The dark red line in (C3) represents a moving average of the stimulation waveform (sliding window corresponding approximately to two fast-population cycles.).

**Figure 9:**
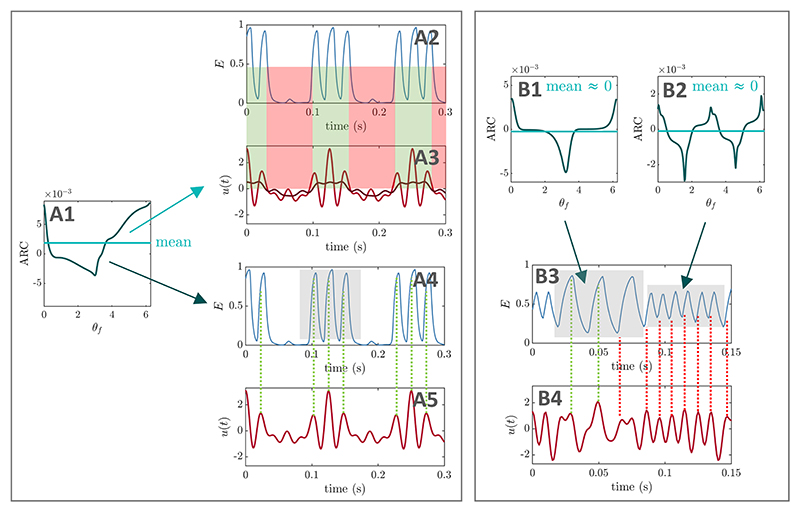
PAC-enhancing mechanisms in the Wilson−Cowan model. (A) corresponds to the strong theta case, and (B) to the pure gamma case. The ARCs shown were calculated in the regimes highlighted in grey. In (A), the amplitude response of the excitatory population depends on its phase and has a non-zero mean (highlighted in light green in (A1)). The optimal stimulation waveform (taken from figure 7(C)) combines the mechanisms of PAC-enhancement corresponding to the foundational cases one and two in the SL model (slow-frequency stimulation in (A2)−(A3), and fast-frequency stimulation in (A4)−(A5)). The dark red line in (A3) represents a moving average of the optimal stimulation waveform (sliding window corresponding approximately to two fast-population cycles). In (B), the amplitude response of the excitatory population has a mean close to zero (light green line in (B1)−(B2)), but a strong phase dependence. Thus, only the mechanism corresponding to foundational case two in the SL model is at play here (fast-frequency stimulation in (B3)−(B4)). The ARC strongly depends on the amplitude of the excitatory population, and the presence of a strong second harmonic in (B2) leads to a strong component around twice the fast frequency in the stimulation waveform.

We will show that systems with different ARCs require different stimulation waveforms to optimally modulate PAC. Examples of ARCs with their corresponding optimal PAC-enhancing waveforms are given for the SL model in figure 2, and later for the WC model in figure 9.

Before dealing with arbitrary stimulation coupling functions (i.e. arbitrary ARCs and PRCs), we consider two foundational cases to uncover the two mechanisms of action contributing to PAC enhancement in the general case. We will show below that in the first foundational case where the amplitude response of the fast population does not depend on its phase, the optimal stimulation is at the frequency of the slow rhythm (figure 2(A)). In the second foundational case where the amplitude response of the fast population depends on its phase but has zero mean, the optimal stimulation is at the fast frequency, with fast frequency components modulated by the slow frequency (figure 2(B)). The general case combines both strategies (figure 2(C)). In each case, we derive theoretical results and test them using numerical optimisation.

#### 2.1.1 Foundational case one: stimulation is coupled through the mean-field

In this first foundational case where stimulation is coupled to the fast population through its mean field, i.e. *f*(*z_f_*) = *z_f_*, the optimal PAC-enhancing waveform can be approximated analytically. Using equation (3), we note that this type of stimulation coupling through the mean-field of the fast population is equivalent to ARC = *ρ_f_* and PRC = 0. The ARC is therefore positive, with no dependence on the fast population phase. We have

(4) $${\dot{z}}_{f}=\left(\delta +k_{s}\text{cos}\omega_{s}t+u(t)+\text{i}\omega_{f}-{\left|z_{f}\right|}^{2}\right)z_{f},$$

and the parameter controlling the amplitude of the fast oscillations is therefore given by *δ*_PAC_ = *δ* + *k_s_* cos*ω_s_ t* + *u*(*t*).

##### 2.1.1.1 Approximate analytical solution

To analytically quantify PAC in the system described by equation (4), we modify a PAC measure called the mean vector length (MVL) [39, 40]. The MVL is recommended for high signal-to-noise ratio [40], which is the case in this modelling approach. We define our modified MVL measure as

(5) $$\text{Γ}=\frac{1}{T_{s}}\left|\int_{0}^{T_{s}}\rho_{f}{\left(t\right)}^{2}{\text{e}}^{\text{i}\omega_{s}t}\text{d}t\right|,$$

where *T_s_* = 2*π/ω_s_* is the period of the slow rhythm, *ρ_f_* is the amplitude of the fast oscillations, and *ω_s_t* is the phase of the slow input. Our PAC measure Γ is the direct translation of the MVL (as defined in [40]) to continuous time over one period, with the exception that the amplitude of the fast oscillations is replaced by *ρ_f_*(*t*)^2^ (i.e. power) for analytical convenience (as will become apparent below). As in the original definition, when the amplitude of the fast oscillation is high for a consistent range of phases of the slow oscillation, the magnitude of the resulting vector will be large and PAC will be detected. Assuming the square of the envelope of the fast oscillations can be expressed as a Fourier series, our PAC measure Γ can also be interpreted as the modulus of the complex Fourier coefficient of $\rho_{f}^{2}$ at the slow frequency. The modified PAC measure Γ therefore captures the strength of the modulation of *ρ_f_* at the slow frequency *ω_s_*.

Assuming relaxation to the limit cycle arising from equation (4) is fast enough, we have $\rho_{f}(t)\approx \sqrt{\delta_{\text{PAC}}(t)}$ for *δ*_PAC_(*t*) > 0, which allows us to compute Γ (this assumption can be relaxed using a semi-analytical approach described in supplementary material section A). We therefore have

$$\begin{array}{l} \text{Γ}=\frac{2\pi }{\omega_{s}}|\int_{0}^{\frac{2\pi }{\omega_{s}}}[\delta +k_{s}\text{cos}\omega_{s}t+\overset{N_{u}}{\underset{n=1}{\sum }}\{a_{n}\text{cos}\left(n\omega_{s}t\right)\\ \;+b_{n}\text{sin}\left(n\omega_{s}t\right)\}]{\text{e}}^{\text{i}\omega_{s}t}\text{d}t|. \end{array}$$

The only non-zero terms correspond to products of sines or cosines at the same frequency, which yields

(6) $$\text{Γ}=\frac{1}{2}{\left[{\left(a_{1}+k_{s}\right)}^{2}+b_{1}^{2}\right]}^{\frac{1}{2}}.$$

Remarkably, the PAC measure Γ only depends on the first harmonic of the stimulation. For a given stimulation energy Ξ, we can find the values of *a*_1_ and *b*_1_ that maximise Γ using the method of Lagrange multipliers. The energy constraint is $a_{1}^{2}+b_{1}^{2}=2\Xi$, and the corresponding Lagrangian function reads

$$ℒ\left(a_{1},b_{1},\lambda \right)=\frac{1}{2}{\left[{\left(a_{1}+k_{s}\right)}^{2}+b_{1}^{2}\right]}^{\frac{1}{2}}+\lambda \left(a_{1}^{2}+b_{1}^{2}-2\Xi \right).$$

Setting its derivatives with respect to *a*_1_, *b*_1_, and λ to 0 leads to $a_{1}=\sqrt{2\Xi }$ and *b*_1_ = 0, i.e.

(7) $$u(t)=\sqrt{2\Xi }\text{cos}\left(\omega_{s}t\right).$$

The optimal stimulation strategy therefore consists in providing sinusoidal stimulation at the slow frequency, with its peak aligned to the peak of the slow rhythm. This optimal waveform makes intuitive sense since the ARC of the fast population is positive and does not depend on the phase of the fast population. Sinusoidal stimulation therefore enhances the amplitude of the fast population when the stimulation waveform is positive, and suppresses the amplitude of the fast population when the stimulation waveform is negative as illustrated in figure 2(A).

##### 2.1.1.2 Verification using numerical optimisation

We verify using numerical optimisation that the waveform given by equation (7) closely approximates the optimal PAC-enhancing waveform. To this end, we optimise the Fourier coefficients of *u*(*t*) up to *N_u_* = 5 to maximise the MVL (obtained as equation (12), see section A.1 in the appendix) while constraining the energy of *u*(*t*) to Ξ. Methodological details of the optimisation process can be found in section A.2.

**Figure 3:**
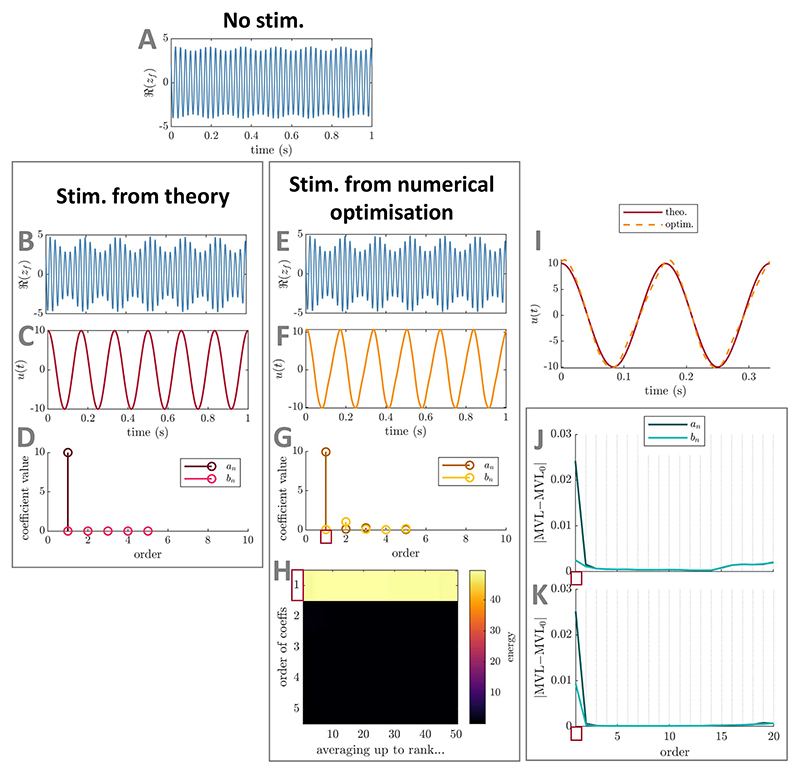
Comparison between optimal PAC-enhancing waveforms predicted by theory and by numerical optimisation—foundational case one (mean-field coupled stimulation) in the Stuart−Landau model. The model output in the absence of stimulation is shown in panel (A). The model output when receiving optimal PAC-enhancing stimulation is shown in panels (B) (stimulation waveform predicted by theory) and (E) (stimulation waveform obtained though numerical optimisation). The corresponding optimal PAC-enhancing stimulation waveforms are shown in panels (C) and (F), respectively, and are overlaid for comparison in panel (I). Their Fourier coefficients are shown in panels (D) and (G), respectively. Panel (H) represents the energy of PAC-enhancing waveforms obtained from numerical optimisation for all Fourier coefficient orders (vertical axis), when averaging the *x*-best optimisation results (*x* being the horizontal axis value). The absolute change in MVL when increasing the energy of a given stimulation Fourier coefficient is provided in panels (J) (when starting from PAC-enhancing waveforms obtained from the numerical optimisation process), and (K) (when starting from random waveforms). Error bars (too small to see here) represent the standard error of the mean. The Fourier coefficients predicted to be key contributors to PAC levels by theory are highlighted by red rectangles in panels (H), (J), and (K). MVL for the stimulation waveform predicted by theory is 0.335, MVL for the stimulation waveform obtained though numerical optimisation is 0.337, MVL in the absence of stimulation is 0.079 (Δ*f_f_* = 10 Hz). In all cases, waveform energy is fixed at Ξ = 50. The parameters of the SL model used are *δ* = 15, *k_s_* = 3, *f_f_* = 40 Hz, and *f_s_* = 6 Hz.

The best-ranked stimulation waveform obtained from numerical optimisation is a close approximation of the sinusoidal waveform given by equation (7), as shown in figure 3 (see panel (I), and compare panels (D) and (G)). These results are consistent across the top-50 optimisations (figure 3(H)). Note that similar results are obtained when maximising the MVL (figure 3) or a discrete approximation of Γ, i.e. measures based on *ρ_f_* or $\rho_{f}^{2}$, respectively. We also perturb each Fourier coefficient in turn by adding the perturbation $\sqrt{\Xi }/10$, where Ξ is the waveform energy before perturbation. This analysis confirms the dominant impact of the first Fourier component of the stimulation waveform on PAC. This is true both when perturbing PAC-enhancing waveforms (figure 3(J)) and random waveforms (figure 3(K)). Methodological details for this analysis can be found in section A.3.

#### 2.1.2 Foundational case two: stimulation acts through a direct coupling

We next consider a second foundational case where stimulation acts through *f*(*z_f_*)= 1, which we call ‘direct’ coupling. With the activity of the fast population modelled as **ℜ**(*z_f_*) (where **ℜ**(.) denotes the real part), this case represents stimulation directly increasing the firing rate of the fast population. Using equation (3), *f*(*z_f_*)= 1 corresponds to ARC = cos*θ*_f_ and PRC = − sin*θ*_f_*/ρ_f_* (for *ρ_f_* > 0). From equation (1) with direct coupling, the time evolutions of *ρ_f_* and *θ*_f_ are given by

(8) $$\begin{array}{l} {\dot{\rho }}_{f}=-\rho_{f}^{3}+\left[\delta +k_{s}\text{cos}\left(\omega_{s}t\right)\right]\rho_{f}+\text{cos}\left(\theta_{f}\right)u(t),\\ {\dot{\theta }}_{f}=\omega_{f}-\frac{\text{sin}\left(\theta_{f}\right)}{\rho_{f}}u(t), \end{array}$$

for *ρ_f_* > 0.

##### 2.1.2.1 Theoretical predictions

While an analytical solution is out of reach, we can determine which Fourier coefficients of the stimulation waveform should be considered to enhance PAC. To study the effect of stimulation on *ρ_f_*, we approximate *θ*_f_ by

(9) $$\theta_{f}\approx \omega_{f}t+\varphi_{u}\approx r\omega_{s}t+\varphi_{u},$$

where *r* is the closest integer to *ω_f_/ω_s_*, and *φ_u_* is a constant phase (the subscript *u* denotes a potential dependence on the stimulation waveform). This approximation is justified if the stimulation amplitude is small (in which case deviations of ${\dot{\theta }}_{f}$ from *ω*_*f*_ would be small), and *ω*_s_ ≫ *ω_s_*, which is often the case in the brain (e.g theta-gamma coupling). The approximation *θ*_f_ ≈ *rω_s_t* + *φ_u_* is also justified for larger stimulation amplitudes leading to *r* : 1 entrainment. We therefore have approximately

(10) $${\dot{\rho }}_{f}=-\rho_{f}^{3}+\left[\delta +k_{s}\text{cos}\left(\omega_{s}t\right)\right]\rho_{f}+\text{cos}\left(r\omega_{s}t+\varphi_{u}\right)u(t).$$

Although an exact solution has recently been found for this type of differential equations (Abel’s equation of the first kind) [41], it cannot be expressed directly as a function of the Fourier coefficients of the stimulation. Instead, we can gain insight by noting that in the steady-state, solutions with PAC will be periodic with period 2*π/ω_s_*, and thus can be approximated as truncated Fourier series. Since most of the PAC strength is captured by the first harmonic of *ρ_f_*, we only consider its zeroth and first order components parametrised by *ρ*_0_, *ρ*_1_, and *θ*_1_ such that *ρ_f_*(*t*) *ρ*_0_ + 2*ρ*_1_ cos(*ω_s_t* + *θ*_1_). We show in section A.4 in the appendix that equation (10) translates to three equations in *ρ*_0_, *ρ*_1_, and *θ*_1_ (equations (15)–(17)).

While these equations cannot be solved easily, they demonstrate that the zeroth and first harmonic of *ρ* (which will determine PAC strength) only depend on the Fourier coefficients of the stimulation of order *r, r* − 1, *r* + 1 (recall that *r* is the closest integer to *ω_f_/ω_s_*). Since the base frequency of *u*(*t*) is *ω_s_*, these Fourier coefficients correspond to frequencies *f_f_*, and *f_f_* ± *f_s_*. The small number of Fourier coefficients involved significantly simplifies the task of finding an optimal stimulation to increase PAC, and also high-lights that how stimulation couples to the neural circuit of interest has a large influence on the optimal stimulation. The optimal stimulation here is very different from the mean-coupling case where stimulation at *ω_s_* is optimal, i.e. only *u*_1_ is non-zero. Note the theory predicts that no Fourier coefficient other than the coefficients of order *r, r* − 1, *r* + 1 plays a key role in enhancing PAC, but the coefficients of order *r, r* − 1, *r* + 1 need not all have a significant impact on PAC.

##### 2.1.2.2 Verification using numerical optimisation

We verify using numerical optimisation that the key stimulation waveform Fourier coefficients to optimally enhance PAC (for direct coupling) are limited to (possibly a subset of) coefficients of order *r, r* − 1, and *r* + 1 as predicted by theory. To this end, we optimise either the Fourier coefficients of *u*(*t*) predicted by theory, or all Fourier coefficients up to *N*_*u*_ = 10. In both cases, the objective is to maximise the MVL (obtained as equation (12), see section A.1 in the appendix) while constraining the energy of *u*(*t*) to Ξ. As before, methodological details of the optimisation process can be found in section A.2.

**Figure 4:**
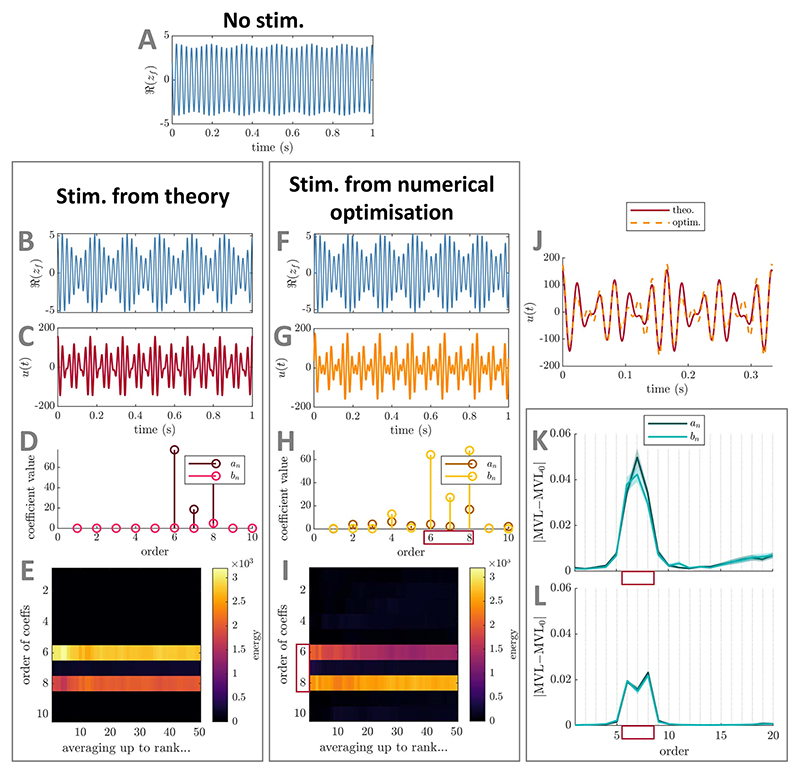
Comparison between best PAC-enhancing waveforms predicted by theory and by numerical optimisation—foundational case two (direct stimulation coupling) in the Stuart−Landau model. The model output in the absence of stimulation is shown in panel (A). The model output when receiving PAC-enhancing stimulation is shown in panels (B) (best stimulation waveform obtained when optimising only Fourier coefficients predicted by theory) and (F) (best stimulation waveform obtained when optimising all Fourier coefficients). The corresponding best PAC-enhancing stimulation waveforms are shown in panels (C) and (G), respectively, and are overlaid for comparison in panel (J) (aligned to maximise their cross-correlation). Their Fourier coefficients are shown in panels (D) and (H), respectively. The energy of PAC-enhancing waveforms obtained from numerical optimisation for all Fourier coefficient orders (vertical axis) when averaging the *x*-best optimisation results (*x* being the horizontal axis value) is represented in panels (E) (only Fourier coefficients predicted by theory were optimised) and (I) (all Fourier coefficients were optimised). The absolute change in MVL when increasing the energy of a given stimulation Fourier coefficient is provided in panels (K) (when starting from PAC-enhancing waveforms obtained from the numerical optimisation process with all coefficients optimised), and (L) (when starting from random waveforms). Error bars represent the standard error of the mean. The Fourier coefficients predicted to be (potential) key contributors to PAC levels by theory are highlighted by red rectangles in panels (H), (I), (K), and (L). MVL for the stimulation waveform with only coefficients predicted by theory optimised is 0.563, MVL for the stimulation waveform with all coefficients optimised is 0.533, MVL in the absence of stimulation is 0.082 (Δ*f_f_* = 20 Hz). In all cases, waveform energy is fixed at Ξ = 5000. The parameters of the SL model used are *δ* = 15, *k_s_* = 3, *f_f_* = 42 Hz, and *f_s_* = 6 Hz (*r* = 7).

The results of numerical optimisation support theoretical predictions. The best-ranked stimulation waveforms obtained from both numerical optimisations show close similarities (figure 4(J)), and the resulting PAC levels are similar (with a slight advantage when optimising Fourier coefficients predicted by theory). Additionally, the best-ranked wave-form obtained when optimising all coefficients concentrates its energy in the Fourier coefficients predicted by theory (highlighted by a red rectangle in figure 4(H)). This is consistent across the top-50 local optimisations (figure 4(I)). Moreover, perturbing individual Fourier coefficients in turn confirms the dominant impact of the stimulation waveform Fourier components of order *r, r* − 1, and *r* + 1. This is true both when perturbing PAC-enhancing waveforms (figure 4(K)) and random waveforms (figure 4(L)). As before, the perturbation size is $\sqrt{\Xi }/10$ and methodological details for this analysis can be found in section A.3. These numerical results were obtained for *ω_f_/ω_s_* = 7, and we also verify that our theoretical predictions hold true for non-integer values of *ω*_f_/*ω_s_* (see figure S.2 in supplementary material).

This second foundational example illustrates a key mechanism of action of PAC-enhancing stimulation when the amplitude response of the fast population depends on its phase and has zero mean. In this case, modulating the amplitude of the fast population necessarily requires fast-frequency oscillations in the stimulation waveform. The optimal waveform obtained from numerical optimisation in figure 2(B) demonstrate that, if the ARC is maximum and positive at *θ*_f_ = 0, the fast-frequency oscillations in the stimulation waveform should phase-align with the oscillations of the fast population in the part of the slow-frequency cycle where the fast-oscillation amplitude should be increased (see green dashed lines in figure 2(B)). Conversely, if the ARC is minimum and negative at *θ*_f_ = *π* for example, the fast-frequency oscillations in the stimulation waveform should anti-phase-align with the oscillations of the fast population in the part of the slow-frequency cycle where the fast-oscillation amplitude should be decreased (see red-dashed lines in figure 2(B)). While the theoretical analysis presented above does not describe how the fast-frequency oscillations in the optimal wave-form should be arranged, the definition of the ARC requires the phase alignment between the stimulation’s fast components and the oscillations of the fast population to change throughout the slow cycle. Indeed, if this were not the case, the effect of stimulation on the amplitude of the fast population would be the same throughout the slow cycle as the peaks of stimulation at the fast frequency would consistently occur around the same phase of the fast population’s oscillation. As detailed above, the optimal waveform obtained from numerical optimisation confirms that the ARC of the fast population dictates how phase alignment between the stimulation’s fast components and the fast rhythm should change throughout the slow cycle to optimally enhance PAC. Specifically, stimulation energy should be concentrated close to the phase corresponding to maximum amplification in the ARC where the fast population’s oscillations should be strengthened, and close to phase corresponding to maximum suppression in the ARC where the fast population’s oscillations should be weakened.

### 2.1.3 Stimulation acts through a general coupling

In this section, we consider that stimulation acts through a general coupling. In general, the ARC of the fast population will combine features of the foundational cases investigated in the previous sections, i.e. non-zero mean and phase dependence. We assume $f\left(z_{f}\right)=\text{ARC}\left(\theta_{f},\rho_{f}\right){\text{e}}^{\text{i}\theta_{f}}+\text{iPRC}\left(\theta_{f},\rho_{f}\right)z_{f}$ where ARC(*θ*_f_, *ρ_f_*) is a separable function of *θ*_f_ and *ρ_f_*. This is for instance the case for the mean-field of a pop-ulation of neurons represented by phase oscillators [42]. Since the ARC of the fast population is also a periodic function of *θ*_f_, it can be approximated as $\text{ARC}\left(\theta_{f},\rho_{f}\right)\approx g\left(\rho_{f}\right)\sum_{n=-N_{a}}^{N_{a}}\alpha_{n}{\text{e}}^{n\text{i}\theta_{f}}.$

#### 2.1.3.1 Theoretical predictions

As before, we aim to determine which Fourier coefficients of the stimulation waveform should be considered to enhance PAC. We show in section A.5 in the appendix that the zeroth and first order components of *ρ_f_* (parametrised by *ρ*_0_, *ρ*_1_, and *θ*_1_ as previously) must satisfy equations (21)−(23), where *g*(*ρ_f_*) was approximated by a truncated Fourier series $g\left(\rho_{f}\right)=\sum_{n=-N_{\gamma }}^{N_{\gamma }}d_{n}\left(\rho_{0},\rho_{1},\theta_{1}\right){\text{e}}^{n\text{i}\omega_{s}t}$ with truncation order *N*_*γ*_ (note that each *d*_*n*_ depends on the Fourier coefficients of *ρ_f_*).

While these equations cannot be solved analytically, they demonstrate that if the ARC of the fast population has *N*_*a*_ Fourier coefficients, the zeroth and first harmonic of *ρ_f_* (and therefore PAC strength) only depend on (possibly a subset of) the Fourier coefficients of the stimulation of order

$$\begin{array}{l} 1,2,\ldots ,N_{\gamma }+1\\ r-N_{\gamma }-1,\ldots ,r+N_{\gamma }+1\\ 2r-N_{\gamma }-1,\ldots ,2r+N_{\gamma }+1\\ ...\\ N_{a}r-N_{\gamma }-1,\ldots ,N_{a}r+N_{\gamma }+1. \end{array}$$

To summarize, a *k*th harmonic in the ARC of the fast population leads to coefficients of frequency $kf_{f}$ and $kf_{f}\pm f_{s}$ in the optimal stimulation waveform for *k* > 0, while a non-zero mean in the ARC results in the addition of the slow frequency *f_s_*. Significant dependence of the ARC on *ρ_f_* requires additional neighbouring frequencies in steps of *f_s_* until *±*(*N*_*γ*_ + 1)*f_s_* from $kf_{f}$, and until (*N*_γ_ + 1)*f_s_* from *f_s_*. In particular, if the ARC of the fast population has a dominant first harmonic and does not depend strongly on *ρ_f_*, it will be sufficient to optimise the Fourier coefficients of the stimulation waveform corresponding to *f_s_*, *f_f_*, and *f_f_* ± *f*_s_ to determine the optimal stimulation strategy. This corresponds to the combination of the two foundational cases presented earlier.

#### 2.1.3.2 Verification using numerical optimisation

To verify these predictions using numerical optimisation, we consider response curves of the fast population with a non-zero mean and two harmonics given by

$$\begin{array}{l} \text{PRC}\left(\theta_{f},\rho_{f}\right)=g\left(\rho_{f}\right)\left[0.2-\text{sin}\left(\theta_{f}\right)+0.7\text{cos}\left(2\theta_{f}\right)\right],\\ \text{ARC}\left(\theta_{f},\rho_{f}\right)=g\left(\rho_{f}\right)\left[0.4+\text{cos}\left(\theta_{f}\right)-0.5\text{sin}\left(2\theta_{f}\right)\right], \end{array}$$

for *g*(*ρ_f_*)= 1 and *g*(*ρ_f_*) = 1/(*ρ_f_* + 0.01). Thus, we verify in the former case that the key stimulation waveform Fourier coefficients contributing to enhancing PAC are limited to (possibly a subset of) coefficients of order 1, *r, r* − 1, *r* + 1, as well as 2*r*− 1, 2*r*, and 2*r* + 1 as predicted (second harmonic in ARC). In the latter case, the set of predicted potential dependences expand to also include coefficients of order 2, *r* − 2, *r* + 2 as well as 2*r*− 2, and 2*r* + 2 (we take *N_γ_* = 1 since *g*(*ρ_f_*) is well described off-stimulation by one harmonic as shown in figure S.3(A) in supplementary material). As before, we optimise either the Fourier coefficients of *u*(*t*) predicted by theory, or all Fourier coefficients up to *N*_*u*_ = 20 to maximise the MVL while constraining the energy of *u*(*t*) to Ξ (see sections A.1 and A.2 for methodological details).

**Figure 5:**
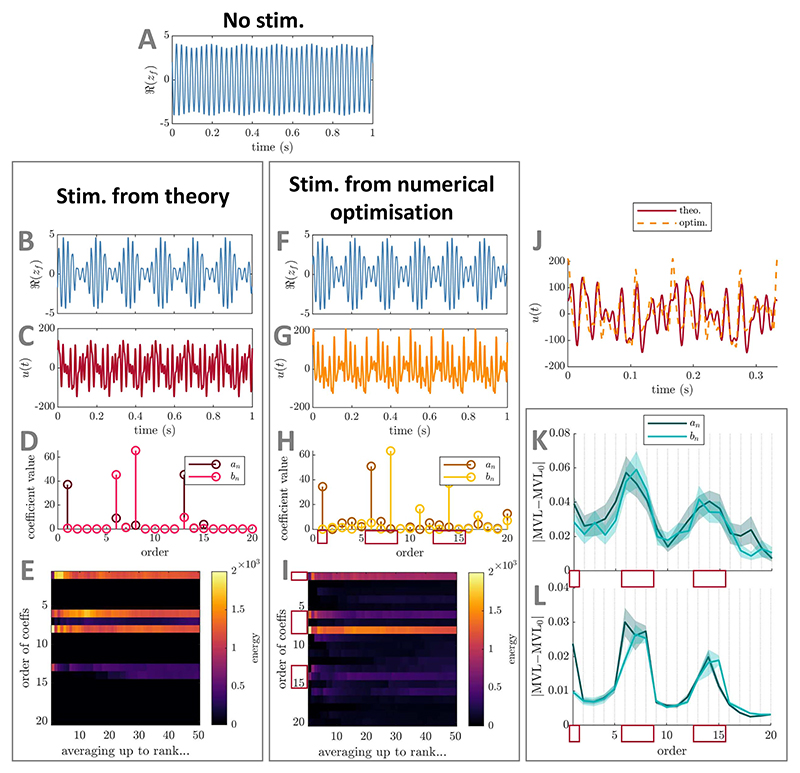
Comparison between best PAC-enhancing waveforms predicted by theory and by numerical optimisation—example for the general stimulation coupling case (no *ρ_f_* dependence) in the Stuart−Landau model. The model output in the absence of stimulation is shown in panel (A). The model output when receiving PAC-enhancing stimulation is shown in panels (B) (best stimulation waveform obtained when optimising only Fourier coefficients predicted by theory) and (F) (best stimulation waveform obtained when optimising all Fourier coefficients). The corresponding best PAC-enhancing stimulation waveforms are shown in panels (C) and (G), respectively, and are overlaid for comparison in panel (J) (aligned to maximise their cross-correlation). Their Fourier coefficients (absolute values) are shown in panels (D) and (H), respectively. The energy of PAC-enhancing waveforms obtained from numerical optimisation for all Fourier coefficient orders (vertical axis) when averaging the *x*-best optimisation results (*x* being the horizontal axis value) is represented in panels (E) (only Fourier coefficients predicted by theory were optimised) and (I) (all Fourier coefficients were optimised). The absolute change in MVL when increasing the energy of a given stimulation Fourier coefficient is provided in panels (K) (when starting from PAC-enhancing waveforms obtained from the numerical optimisation process with all coefficients optimised), and (L) (when starting from random waveforms). Error bars represent the standard error of the mean. The Fourier coefficients predicted to be (potential) key contributors to PAC levels by theory are highlighted by red rectangles in panels (H), (I), (K), and (L). MVL for the stimulation waveform with only coefficients predicted by theory optimised is 0.679, MVL for the stimulation waveform with all coefficients optimised is 0.643, MVL in the absence of stimulation is 0.082 (Δ*f_f_* = 20 Hz). In all cases, waveform energy is fixed at Ξ = 5000. The parameters of the Stuart−Landau model used are *δ* = 15, *k_s_* = 3, *f_f_* = 42 Hz, and *f_s_* = 6 Hz (*r* = 7). Stimulation is acting through PRC(*θ*) = 0.2 − sin(*θ*) + 0.7 cos(2*θ*) and ARC(*θ*) = 0.4 + cos(*θ*) − 0.5 sin(2*θ*).

In both cases, the results of numerical optim-isation support theoretical predictions (see figure 5 for *g*(*ρ_f_*) = 1 and figure S.4 for *g*(*ρ_f_*) = 1/(*ρ_f_* + 0.01) in supplementary material). The best-ranked stimulation waveforms obtained from optimising coefficients predicted by theory and all coefficients show similarities (panels (J) in both figures), and the resulting PAC levels are similar (in both cases with a slight advantage when optimising Fourier coefficients predicted by theory). Additionally, the best-rank waveforms obtained when optimising all coefficients concentrate their energy in a subset of the Fourier coefficients predicted by theory (highlighted by red rectangles in panels (H) in both figures). In both cases, this is consistent across the top-50 local optimisations (panels (I) in both figures), and confirmed by perturbation analysis (perturbation of PAC-enhancing waveforms in panels (K) and of random waveforms in panels (L), see section A.3 for methodological details, perturbation of size $\sqrt{\Xi }/10$ as before). Comparing figures 5(H) and S.4(H), significant energy is introduced in Fourier coefficients of order 2 and 2*r* − 1 when the ARC of the fast population depends on *ρ_f_* as opposed to when it does not. However the Fourier components with the largest impact on PAC are the same in both cases (see panels (K) and (L)). In figure S.4(H), the energy of *b*_3_ is not negligible, indicating that *g*(*ρ_f_*)= 1/(*ρ_f_* + 0.01) is best described by two harmonics when stimulation is on (see figure S.3(B2) in supplementary material).

The optimal waveforms obtained from numerical optimisation in figures 5(C) and S.4(C) combine the two PAC-enhancing mechanisms presented in the two foundational cases (see figures 2(A) and (B)). Because the mean amplitude response of the fast population across phases is non-zero (figure 2(C1)), the optimal stimulation waveform has a slow-frequency component that directly participates in expanding and shrinking the fast-frequency oscillations to produce PAC (figures 2(C2)−(C3) and S.5(C)−(D) in supplementary material). Moreover, because the amplitude response of the fast population strongly depends on its phase (figure 2(C1)), modulating the amplitude of the fast population requires fast-frequency components in the stimulation waveform whose alignment with the fast rhythm is modulated throughout the slow cycle (figures 2(C4)−(C5) and S.5(E)−(F) in supplementary material).

Using the SL model, we have identified how characteristics of the fast population’s response to stimulation dictate which frequencies should be included in the optimal stimulation waveform, and how to align potential fast stimulation components throughout the slow cycle according to the fast population’s ARC. We next investigate whether these predictions carry over to a neural mass model generating PAC.

### 2.2 Testing optimal PAC-enhancing stimulation in the Wilson−Cowan model

**Figure 6:**
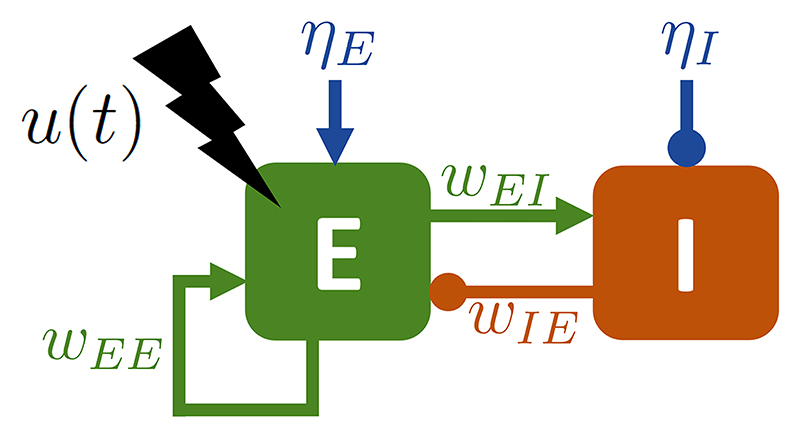
Sketch of the Wilson−Cowan model with stimulation. Intrinsic PAC can be generated by a slow oscillatory input *㮇_E_* provided to the excitatory population (denoted *E* and shown in green). The inhibitory population (denoted *I* and shown in red) receives a constant input *㮇_I_*. The excitatory and inhibitory populations are reciprocally coupled, and the excitatory population has a self-excitatory connection. The stimulation *u*(*t*) (in black) acts on the excitatory population.

To test predictions obtained with the SL model, we use a more realistic neural mass model representing interacting neural populations, the Wilson–Cowan model [43]. This model was proposed as a canonical circuit to generate theta-gamma PAC in the presence of a theta input [44]. The biologically-inspired WC model describes the interactions of an excitatory (E) and an inhibitory (I) population (see figure 6). The model is presented in details in section A.6 in the appendix.

**Figure 7:**
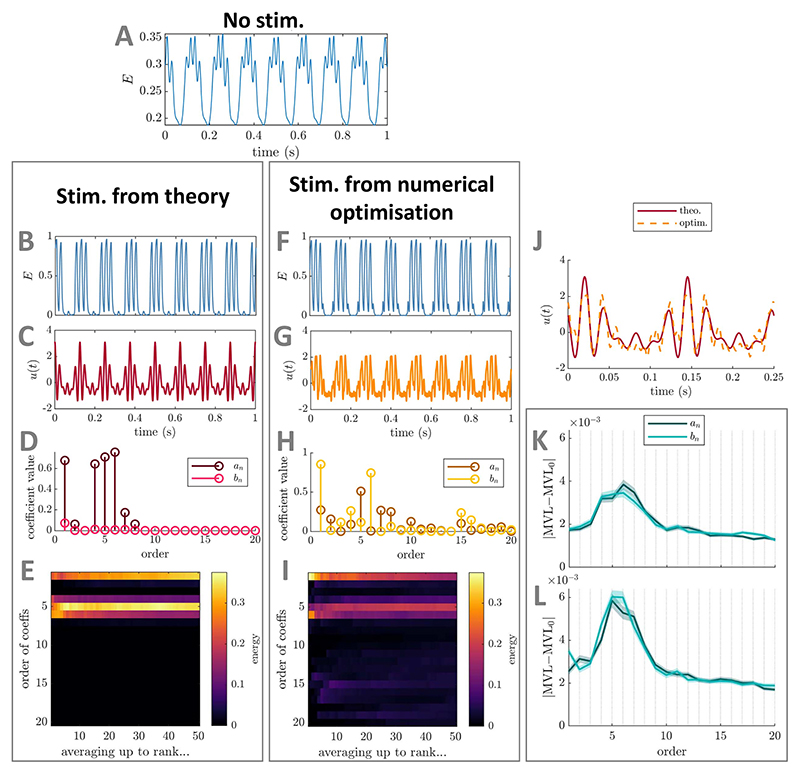
Comparison between best PAC-enhancing waveforms predicted by theory and by numerical optimisation—strong theta case in the Wilson-Cowan model. The model output in the absence of stimulation is shown in panel (A). The model output when receiving PAC-enhancing stimulation is shown in panels (B) (best stimulation waveform obtained when optimising only Fourier coefficients predicted by theory) and (F) (best stimulation waveform obtained when optimising all Fourier coefficients). The corresponding best PAC-enhancing stimulation waveforms are shown in panels (C) and (G), respectively, and are overlaid for comparison in panel (J) (aligned to maximise their cross-correlation). Their Fourier coefficients are shown in panels (D) and (H), respectively. The energy of PAC-enhancing waveforms obtained from numerical optimisation for all Fourier coefficient orders (vertical axis) when averaging the *x*-best optimisation results (*x* being the horizontal axis value) is represented in panels (E) (only Fourier coefficients predicted by theory were optimised) and (I) (all Fourier coefficients were optimised). The absolute change in MVL when increasing the energy of a given stimulation Fourier coefficient is provided in panels (K) (when starting from PAC-enhancing waveforms obtained from the numerical optimisation process with all coefficients optimised), and (L) (when starting from random waveforms). Error bars represent the standard error of the mean. MVL for the stimulation waveform with only coefficients predicted by theory optimised is 0.102, MVL for the stimulation waveform with all coefficients optimised is 0.101, MVL in the absence of stimulation is 0.0045 (Δ*f_f_* = 20 Hz). In all cases, waveform energy is fixed at Ξ = 1. The parameters of the Wilson−Cowan model used are taken given in table 1 (strong theta row), and *r* = 6 (off-stimulation).

**Figure 8:**
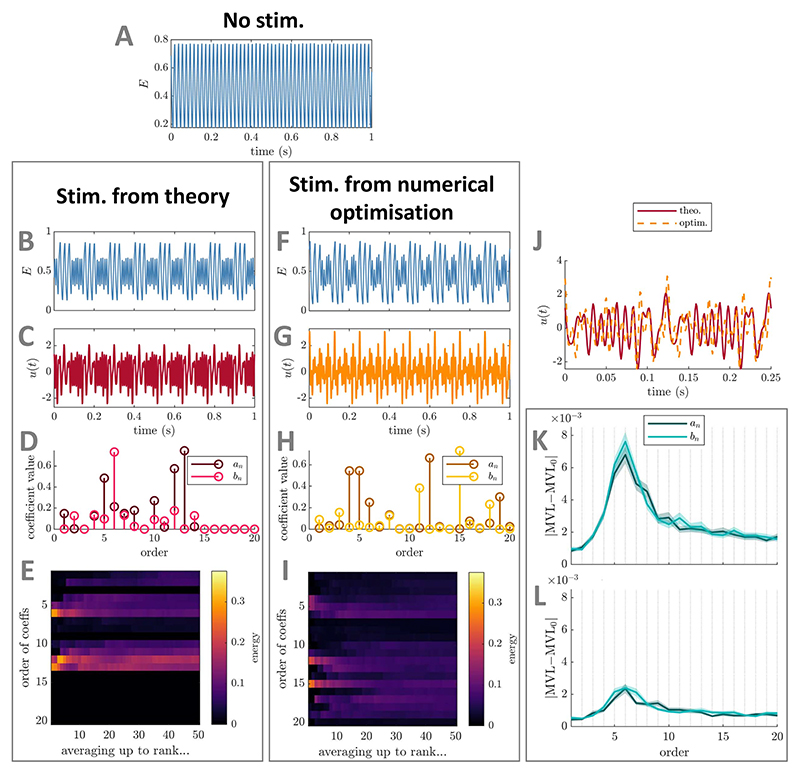
Comparison between best PAC-enhancing waveforms predicted by theory and by numerical optimisation—pure gamma case in the Wilson-Cowan model. The model output in the absence of stimulation is shown in panel (A). The model output when receiving PAC-enhancing stimulation is shown in panels (B) (best stimulation waveform obtained when optimising only Fourier coefficients predicted by theory) and (F) (best stimulation waveform obtained when optimising all Fourier coefficients). The corresponding best PAC-enhancing stimulation waveforms are shown in panels (C) and (G), respectively, and are overlaid for comparison in panel (J) (aligned to maximise their cross-correlation). Their Fourier coefficients are shown in panels (D) and (H), respectively. The energy of PAC-enhancing waveforms obtained from numerical optimisation for all Fourier coefficient orders (vertical axis) when averaging the *x*-best optimisation results (*x* being the horizontal axis value) is represented in panels (E) (only Fourier coefficients predicted by theory were optimised) and (I) (all Fourier coefficients were optimised). The absolute change in MVL when increasing the energy of a given stimulation Fourier coefficient is provided in panels (K) (when starting from PAC-enhancing waveforms obtained from the numerical optimisation process with all coefficients optimised), and (L) (when starting from random waveforms). Error bars represent the standard error of the mean. MVL for the stimulation waveform with only coefficients predicted by theory optimised is 0.069, MVL for the stimulation waveform with all coefficients optimised is 0.070, MVL in the absence of stimulation is 1.3 *×* 10^−5^ (Δ*f_f_* = 20 Hz). In all cases, waveform energy is fixed at Ξ = 1. The parameters of the Wilson−Cowan model used are taken given in table 1 (pure gamma), and *r* = 6 (off-stimulation).

We test our predictions using two dynamically distinct cases. The first is a theta-dominant example with some theta-gamma PAC in the absence of stimulation (figure 7(A)) based on the parameters used in [44] (values given in table 1 in the appendix). This case is inspired by situations where gamma is locked to the peak of theta (e.g. in the human hippocampus during memory encoding [6]), and increasing PAC could be beneficial (e.g in AD). In this case, the WC is in a fixed-point regime when the slow input is low, and crosses the Hopf bifurcation to the limit-cycle regime (gamma oscillations) when the slow input increases (see [44] for more details). We call this example the ‘strong theta case’. Our second case corresponds to a hypothetical scenario where PAC has almost completely disappeared due to pathology and should be restored externally by stimulating the fast rhythm. This second example displays pure gamma oscillations (limit-cycle regime) with no slow input and no PAC in the absence of stimulation (see figure 8(A), parameters in table 1 in the appendix). We call this example the ‘pure gamma case’. To investigate whether predictions from the theory developed using the SL model in section 2.1 carry over to the WC model, we optimise Fourier coefficients of *u*(*t*) up to *N_u_* = 20 under energy constraint for both the strong theta case and the pure gamma case. Methodological details of the optimisation process can be found in section A.2, and methodological details of the perturbation analysis can be found in section A.3 (perturbation size is as before).

To test the predictions of our framework, we also compute the ARC in both cases (methodological details can be found in section A.7 in the appendix). In general, the ARC may depend on the full state of the system (we investigated the case of the dependence on *ρ_f_* in the SL model in section 2.1.3), so for the two-dimensional WC model the ARC may also depend on the amplitude of the oscillations in addition to their phase. Here, we compute the ARC along representative trajectories selected to reflect the dynamical regimes where significant levels of stimulation are provided. For the strong theta case, this corresponds to the high amplitude regime highlighted in gray in figure 9(A4). Stimulation is very low during the adjacent low amplitude regime so the ARC there is not relevant (in this case it is approximately a scaled version of the ARC in the high amplitude regime). For the pure gamma case, we compute the ARC for the two distinct dynamical regimes where significant levels of stimulation are provided: a high amplitude regime (left gray rectangle in figure 9(B3)), and a lower amplitude, higher frequency regime (right rectangle in figure 9(B3)). The details of the trajectories used to compute the ARC are shown in figure S.6 in supplementary material.

In the strong theta case, when optimising all Fourier coefficients of the stimulation waveform, energy is concentrated in coefficients of order 1, *r* − 1, and *r* (see figures 7(H) and (I), *r* = 6 based on off-stimulation frequencies). Perturbation analysis highlights the role of coefficients of order *r* − 2 to *r* + 2 when starting from optimised waveforms, and of order *r* − 1 to *r* + 1 when starting from random waveforms. When only optimising coefficients of order 1, 2, and *r* − 2 to *r* + 2, we find significant energy only in coefficients of order 1 and *r* − 2 to *r* (figures 7(D) and (E)), and a slightly more favorable MVL value than when optimising all coefficients (see figure caption). In light of the ARC of the stimulated WC population, these results are consistent with the predictions obtained previously. In particular, the ARC has a non-zero mean (light green line in figure 9(A1)), which gives leverage to the Fourier coefficients of the stimulation waveform at the slow frequency, i.e. of order 1 (figures 9(A2) and (A3)). Furthermore, the phase-dependence of the ARC is described by a strong first harmonic (with some dependence on *ρ_f_*), hence the key role played by coefficients belonging to orders *r* − 2 to *r* + 2 (no strong involvement of coefficients at the second harmonic). Both mechanisms of actions of optimal PAC-enhancing waveforms identified in the SL model are therefore preserved in this example (figures 9(A2)−(A5)).

In the pure gamma case, the involvement of stimulation waveform Fourier coefficients around the second harmonic of the fast frequency is much more pronounced, and the slow frequency is absent. When optimising all coefficients, energy is concentrated in coefficients of order *r* − 2 to *r*, and 2*r* to 2*r* + 3 (see figures 8(H) and (I), *r* = 6 based on off-stimulation frequencies). Perturbation analysis underlines the impact of coefficients of order *r* − 1 to *r* + 1 (figures 8(K) and (L)). When only optimising coefficients of order 1, 2, *r* − 2 to *r* + 2, and 2*r*− 2 to 2*r* + 2, we find significant energy only in coefficients of order *r* − 2 to *r*, and 2*r* − 2 to 2*r* + 1 (figures 8(D) and (E)), and a slightly less favorable MVL value than when optimising all coefficients (see figure caption). Given the ARC of the stimulated WC population, these findings align with prior predictions. Since the ARC mean is close to zero (light green lines in figures 9(B1) and (B2)), optimal stimulation waveforms have no significant energy at the slow frequency. Moreover, the ARC shows a strong first harmonic when *ρ_f_* is high (figure 9(B1)). When *ρ_f_* is low, the frequency of the fast oscillations doubles (faster frequency associated with the unstable fixed point enclosed by the limit cycle), which corresponds to a dominant second harmonic in the ARC (figure 9(B2)). According to the previously developed theory and given the dependence on *ρ_f_*, this cor-responds to the potential involvement of stimulation coefficients of order *r* − 2 to *r* + 2, and 2*r*− 2 to 2*r* + 2, which is verified here. An exception to this is the 2*r* + 3 term seen in figure 8(I), which may be due to the speed-up of fast oscillations at low *ρ_f_* (*r* = 5 on stimulation), or to the fact that the dependence on *ρ_f_* cannot be described sufficiently well by a single harmonic.

### 2.3 Practical considerations

**Figure 10:**
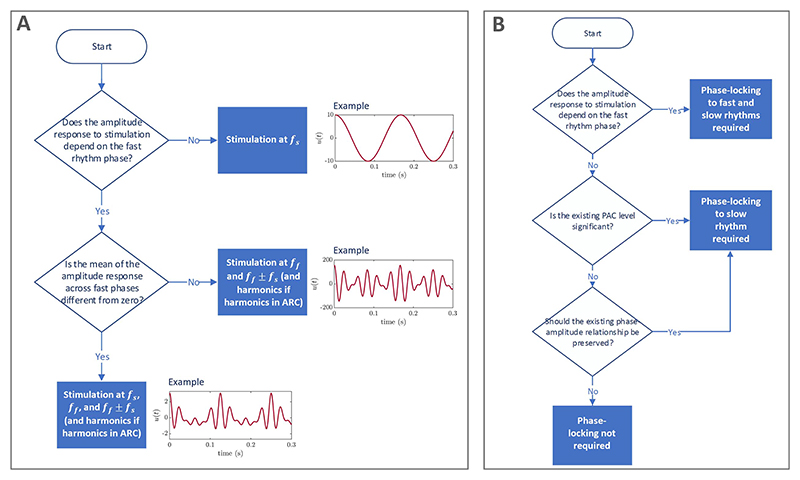
Simplified flowcharts to guide the design of optimal PAC-enhancing stimulation. We are assuming that stimulation acts solely on the fast population. The flowchart in panel (A) presents which Fourier coefficients of the stimulation waveform to optimise. Neighbouring frequency components may be added if the result is not satisfactory, potentially indicating a dependence of the amplitude response on the amplitude of the fast population. The flowchart in panel (B) assumes that stimulation does not significantly entrain the slow rhythm and presents a guide to decide whether phase-locking the stimulation to the fast and/or slow rhythms is necessary.

We begin by summarising in a flowchart (figure 10(A)) the insights from the previous sections with a view to help experimentalists design PAC-enhancing stimulation. As a reminder, we are assuming that stimulation solely affects the fast population, and our predictions are based on the ARC of the fast population (representing the change in the amplitude of the fast population as a function of the phase of stimulation), which can be measured experimentally [35, 45]. If the amplitude response to stimulation of the fast rhythm does not depend on its phase, optimal stimulation is at the slow frequency *f_s_*. If the amplitude response does depend on the phase of the fast rhythm and its mean is negligible, then the optimal stimulation is a combination of the fast frequency *f_f_*, as well as *f_f_* ± *f_s_* (and corresponding harmonics if there are strong harmonics in the ARC of the fast population). Otherwise, the optimal stimulation is a combination of these two strategies. Neighbouring frequency components may be added if the result is not satisfactory, potentially indicating a dependence of the amplitude response on the amplitude of the fast population. We also note that the same framework applies if one aims to reduce rather than enhance PAC. The resulting optimal stimulation waveforms will however be different (for example, the waveform will be anti-phase in the case of slow-frequency stimulation). We next examine the trade-offs between Fourier coefficients of optimal PAC-enhancing stimulation waveforms, investigate whether phase-locking to the slow and/or fast rhythms is necessary, and how to approximate optimal PAC-enhancing stimulation waveforms using pulses.

### 2.3.1 Trade-offs between Fourier coefficients of the stimulation waveform

The theory developed in section 2.1 identifies which Fourier coefficients should be considered to build an optimal PAC-modulating stimulation waveform, but does not prescribe how much energy should be assigned to these coefficients (except in the simplest case of stimulation being coupled through the mean-field of the fast population, where only one Fourier component is involved). From the mechanism illustrated in figure 2(B), the waveform can be manually designed such that where the amplitude of the fast oscillation should be increased, the phase alignment between the fast component in the stimulation waveform and the fast rhythm correspond to the maximum amplification in the ARC. Conversely, where the amplitude of the fast oscillation should be decreased, the phase alignment between the fast component in the stimulation waveform and the fast rhythm should correspond to the maximum suppression in the ARC. Here, we investigate numerically whether other principles can be found to guide the design of optimal PAC-modulating stimulation waveforms.

In particular, we aim to contrast optimal PAC-enhancing stimulation for different levels of endogenous fast oscillations and slow input. To this end, we consider the SL model (equations (1) and (3)), with stimulation coupled to the fast population through ARC(*θ*_f_)= 0.5 + cos(*θ*_f_) and PRC(*θ*_f_)= sin(*θ*_f_) for simplicity. As previously outlined, the level of endogenous fast oscillations is controlled in this model by *δ*, and the level of slow oscillations is controlled by *k_s_*.

To efficiently optimise the stimulation waveform across combinations of *δ* and *k_s_*, we simplify the optimisation problem as follows. We parametrise the stimulation waveform Fourier series as

(11) $$u(t)=\sum_{n=1}^{N_{u}}A_{n}\text{cos}(n\omega_{s}t-{\text{Φ}}_{n}),$$

where the amplitudes *A*_*n*_ are positive, and the phases Φ_*n*_ are in [0, 2*π*). In the SL model considered, only Fourier coefficients of order 1, *r* − 1, *r*, and *r* + 1 sig-nificantly impact PAC levels (see section 2.1.3 with *N*_*α*_ = 1 and *g*(*ρ_f_*) = 1). We therefore perform a parameter sweep for combinations of *A*_1_, *A_r−1_*, and *A_r_* (*A*_r+1_ is obtained by matching the target stimulation energy, i.e. $A_{r+1}=\sqrt{2\Xi -A_{1}^{2}-A_{r-1}^{2}-A_{r}^{2})}$. For each combination of amplitudes, we only need to optimise the phases Φ_1_, Φ_*r*−1_, Φ_*r*_, and Φ_*r*+1_. These are simpler optimisations than the method described in section A.2 because there is no non-linear constraint to enforce by the optimiser, and there are only four parameters to optimise. Because of the coarse-graining of amplitudes, this approach is less precise than the full optimisations performed previously, but is more efficient. This allows us to explore changes in optimal stimulation waveform as a function of the strength of endogenous fast and slow oscillations in the model. We use 10 equally spaced amplitude values for each of *A*_1_, *A_r−1_*, and *A_r_* (i.e. 1000 amplitude combinations), and perform five local optimisations per combination. Other methodological details are unchanged and per section A.2 in the appendix.

**Figure 11:**
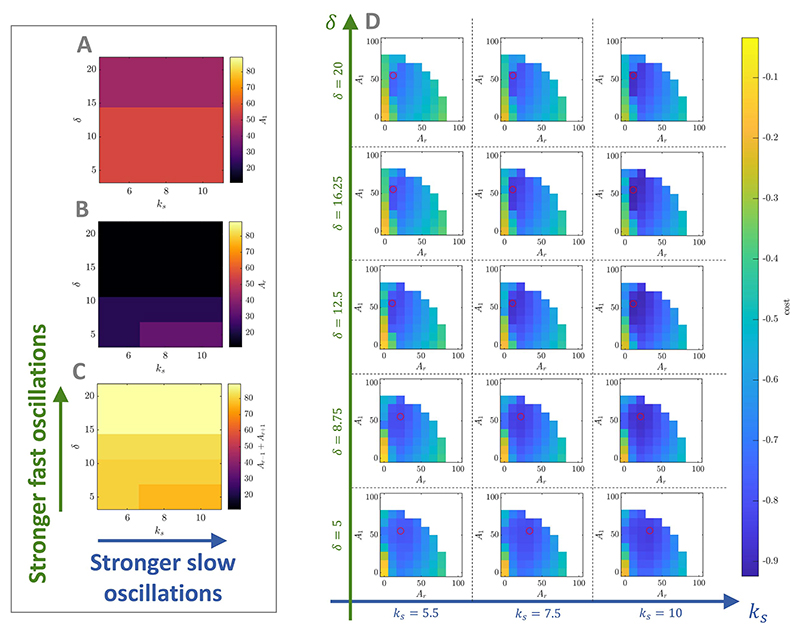
Balance between optimal Fourier amplitudes and cost landscape as a function of the strength of endogenous fast and slow oscillations in the Stuart−Landau model. The strength of endogenous slow oscillations is controlled by model parameter *k_s_* (blue arrows), and the strength of endogenous fast oscillations by model parameter *δ* (green arrows). The balance between Fourier amplitudes of PAC-enhancing optimal stimulation waveforms as a function of the strength of endogenous fast and slow oscillations is shown in panels (A)−(C). Panel (A) corresponds to the slow-frequency Fourier amplitude (*A*_1_), panel (B) to the fast-frequency Fourier amplitude (*A_r_*), and panel (C) to the modulation of the fast frequency at the slow frequency (*A_r−1_* + *A*_r+1_). Panel (D) shows in color the best objective function values (costs) resulting from optimising Fourier phases to enhance PAC for *A_r−1_* = 55.56, as a function of *A*_1_ and *A_r_*, and as a function of the strength of endogenous fast and slow oscillations (specific values indicated on the blue and green axes). For a given combination of *k_s_* and *δ*, the minimum cost for the *A_r−1_* slice shown is highlighted by a red circle. In all panels, the total stimulation waveform energy is kept at Ξ = 5000, and the frequency of endogenous oscillations is *f_f_* = 42 Hz, and *f_s_* = 6 Hz. Stimulation is coupled to the fast population through ARC(*θ*_f_) = 0.5 + cos(*θ*_f_) and PRC(*θ*_f_) = sin(*θ*_f_).

Changes in the balance between Fourier amplitudes as a function of the strength of endogenous fast and slow oscillations in the model (figures 11(A)– (C)) are relatively minor but can be explained intuitively. As endogenous fast oscillations become stronger (increase in *δ*), the Fourier amplitudes corresponding to the fast frequency (*A_r_*) and the slow frequency (*A*_1_) decrease in favor of the Fourier amplitudes corresponding to the modulation of the fast frequency at the slow frequency (*A_r−1_* and *A*_r+1_). There is relatively less endogenous modulation, so an increase in the modulation of the fast frequency is necessary to bring down the trough of *ρ_f_* as per the mechanism described in foundational case two (section 2.1.2 and figure 2(B)). Conversely, provided that endogenous fast oscillations are relatively weak (low *δ*), as endogenous slow oscillations become stronger (increase in *k_s_*), the Fourier amplitudes corresponding to the modulation of the fast frequency at the slow frequency (*A_r−1_* and *A*_r+1_) decrease in favor of the Fourier amplitude corresponding to the fast frequency (*A_r_*). Because endogenous modulation relative to fast-frequency activity is already high, less modulation is needed from stimulation and boosting the fast frequency is advantageous. We choose the model parameters studied in figures 11(A)–(C) to cover a broad range of relative strength of fast to slow oscillations (model output in the absence of stimulation across parameters shown in figure S.7 in the supplementary material). The optimal PAC-enhancing waveforms resulting from the optimisations, as well as the on-stimulation model outputs are shown in figures S.8 and S.9 in the supplementary material, respectively. While small changes in the balance of Fourier amplitudes cannot be detected due to coarse-graining, examination of the cost (here − MVL) across Fourier amplitudes (figure 11(D)) supports the convergence to a single local minimum (for a given set of model parameters). Furthermore, the shift of the entire area of low cost in the space of Fourier amplitudes confirms the trends described above. Figure 11(D) is given for *A_r−1_* = 55.56, but similar results are obtained across all *A_r−1_* considered in the sweep.

### 2.3.2 Is phase-locking to the slow rhythm necessary?

In the theory and examples presented in this work, stimulation is provided with a period corresponding to the slow-oscillation frequency. Thus, stimulation is phase aligned to the slow rhythm at steady-state, and the specifics of the phase alignment are dictated by the Fourier component make-up of the stimulation waveform. With practical applications in mind, we investigate in this section whether phase-locking to the slow-rhythm is necessary to modulate PAC.

To this end, we simulate the SL model with stimulation coupled to the fast population through its mean-field (equation (4), see section 2.1.1). We already know from the analytical analysis in section 2.1.1 that the optimal stimulation is a sinusoid with its peak aligned to the peak of the slow rhythm. However it is unclear how essential this optimal phase alignment is. To answer this question, we provide the optimal PAC-enhancing stimulation at various phases of the slow rhythm and measure resulting PAC levels. We also perform the same analysis in the SL model with stimulation directly coupled to the fast population (as in section 2.1.2), thereby investigating the two foundational cases presented in this work.

**Figure 12:**
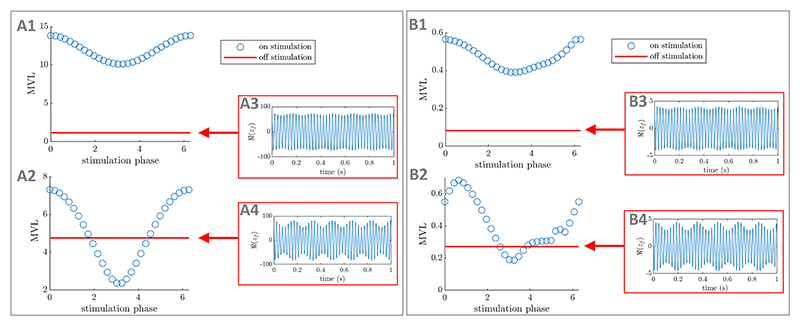
The importance of phase alignment between stimulation and the slow rhythm depends on off-stimulation PAC levels. PAC-enhancing stimulation waveforms are provided at different phases of the slow rhythm. Panels (A1)−(A4) correspond to the SL model with stimulation coupled to the fast population through its mean-field and the stimulation waveform given in figure 3(C), while panels (B1)−(B4) correspond to the SL model with direct stimulation coupling and the stimulation waveform given in figure 4(C). Panels (A1), (A2) and (B1), (B2) show the MVL as a function of stimulation phase of the slow rhythm in blue (a stimulation phase of zero corresponds to the peaks of the stimulation waveform and the slow rhythm being aligned), and the off-stimulation MVL level in red. Panels (A3), (A4) and (B3), (B4) represent the corresponding off-stimulation model output (real part of the order parameter). In (A1)−(A4), *f_f_* = 40 Hz, *f_s_* = 6 Hz, and *δ* = 5000. Panels (A1), (A3) correspond to *k_s_* = 500, and Ξ = 1 *×* 10^7^. Panels (A2), (A4) correspond to *k_s_* = 2000, and Ξ = 5 × 10^5^. In (B1)−(B4), *f_f_* = 42 Hz, *f_s_* = 6 Hz, *δ* = 15, and Ξ = 5000. Panels (B1), (B3) correspond to *k_s_* = 3, and panels (B2), (B4) to *k_s_* = 10. Note that the stimulation waveform provided in (B) is optimal for (B1) but not for (B2).

The importance of phase alignment between stimulation and the slow rhythm depends on PAC levels in the absence of stimulation in both cases. When off-stimulation PAC levels are low (figures 12(A3) and (B3)), a significant PAC-enhancing effect can still be achieved without phase alignment (figures 12(A1) and (B1)). However, when off-stimulation PAC levels are higher (figures 12(A4) and (B4)), providing stimulation close to the optimal phase is critical to enhance PAC (figures 12(A2) and (B2)). For example, providing stimulation half a period too late/early leads to a significant decrease in PAC. We note that the fast-frequency oscillations can be entrained by stimulation in the direct coupling case, but not in the mean-field coupling case (because the PRC is zero). Regardless of the stimulation coupling function, our simulations assume that stimulation does not affect the slow rhythm, and therefore cannot entrain it. Generally, enhancing the existing phase-amplitude relationship between two rhythms requires phase locking, but may be more physiological. This is the case in figures 12(A2) and (B2) where overriding the existing phase-amplitude relationship would require too much energy, and enhancing the existing phase-amplitude relationship is the only viable strategy. Conversely, a phase-amplitude relationship different from the existing phase-amplitude relationship between the fast and slow rhythms is enforced by stimulation for a stimulation phase of e.g. π in figures 12(A2) and (B2).

### 2.3.3 Is phase-locking to the fast rhythm necessary?

In situations where stimulation acts through the mechanism described in foundational case two (see figure 2(B)), the differential alignment (as prescribed by the ARC) of the stimulation fast frequency components with the fast rhythm at the peak and trough of the slow rhythm is critical. In the ideal case where *f_f_* is constant and an integer multiple of *f_s_*, this alignment is enforced as a consequences of phase-aligning stimulation with the slow rhythm. However, we show that in the more realistic scenario where *f_f_* is not an integer multiple of *f_s_* or significantly varies over time, adapting stimulation to the frequency fluctuations of the fast rhythm will give better results. To this end, we simulate the SL model as in the previous section (stimulation coupling corresponding to foundational case two) for different values of *f_s_*, as well as with *f_f_* varying according to a Wiener process.

**Figure 13:**
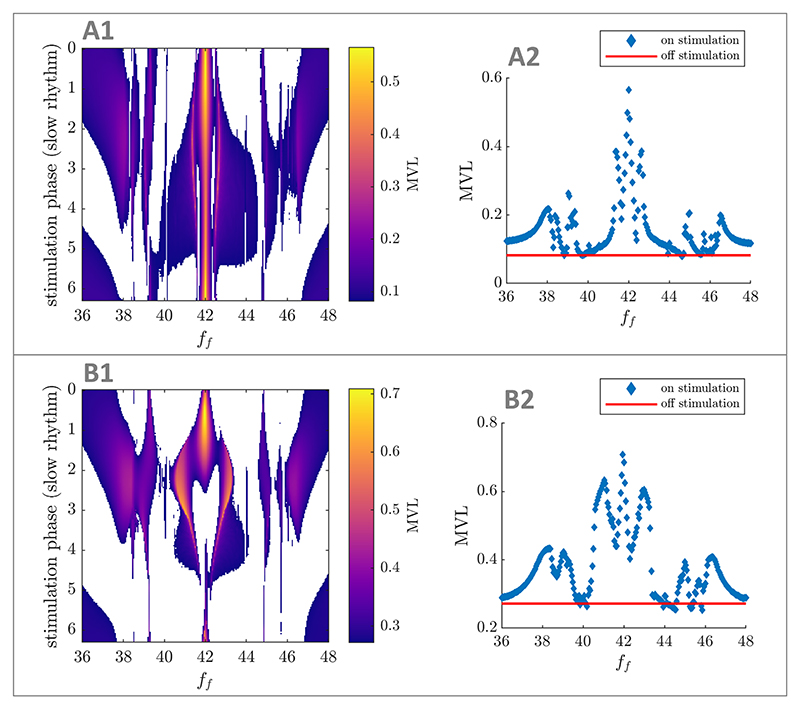
Effect of changes in the fast rhythm frequency on PAC modulation. The PAC-enhancing stimulation waveform optimised for *f_s_* = 6 Hz and *f_f_* = 42 Hz was provided at different phases of the slow rhythm, and for different values of the fast rhythm frequency. Panels (A1) and (B1) show the MVL as a function of *f_f_*, and of the stimulation phase of the slow rhythm (a stimulation phase of zero corresponds to the peaks of the stimulation waveform and the slow rhythm being aligned). No color is shown when the MVL is below the off-stimulation value. The optimal stimulation phase relative to the slow rhythm depends on *f_f_*, and the maximum achievable MVL decreases away from *f_f_* = 42 (in the absence of adjustment of the stimulation to the fast rhythm). This is confirmed in panels (A2) and (B2), where the maximum achievable MVL for a given value of *f_f_* is shown on the vertical axis (off-stimulation MVL level in red). In all panels, simulations are performed using an SL model with direct stimulation coupling and the stimulation waveform given in figure 4(C). Parameters used are *f_s_* = 6 Hz, *δ* = 15, and Ξ = 5000. Panels (A1)−(A2) correspond to *k_s_* = 3, and panels (B1)−(B2) to *k_s_* = 10.

If the stimulation waveform (optimised for *f_f_* = 42 Hz) does not change, the maximum achievable PAC modulation decreases as *f_f_* is varied from 42 Hz (figure 13). The optimal phase alignment between the stimulation waveform and the slow rhythm also changes (figures 13(A1) and (B1)). Similarly, we show that when *f_f_* varies according to a Wiener process, increasing the level of noise drastically reduces the ability of open-loop stimulation to modulate PAC (figure S.10 in supplementary material). Together, these results suggest that if the stimulation waveform contains fast frequency components, it is advisable to lock these with the fast rhythm (in the manner prescribed by the ARC of the fast rhythm). We summarise the conclusions of this subsection and the previous one in the flowchart presented in figure 10(B).

## 2.4 Pulsatile waveforms

**Figure 14:**
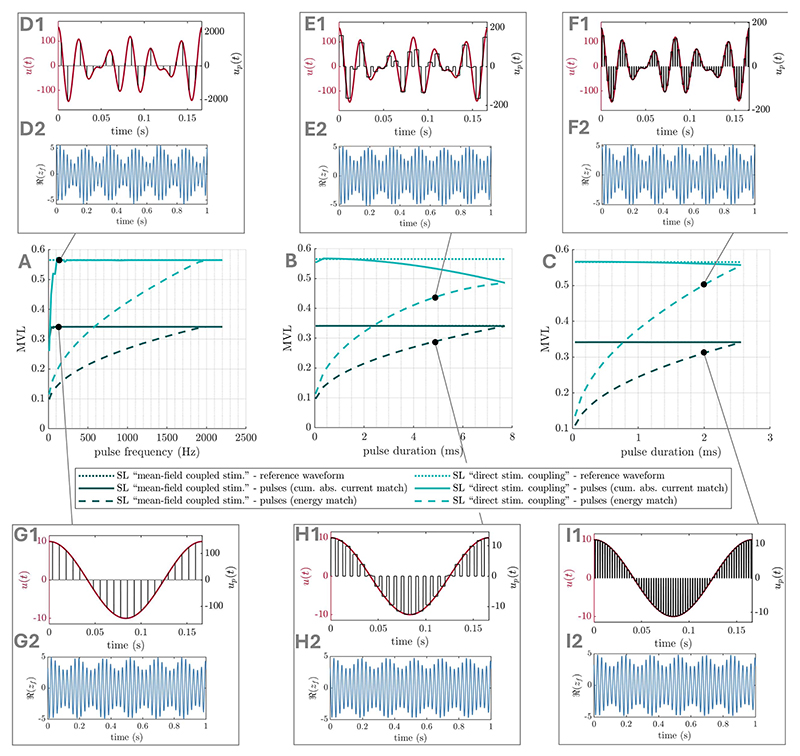
Optimal smooth waveforms can be approximated with pulses—foundational cases one and two in the SL model. MVL is shown as a function of pulse frequency, for a pulse duration of 0.5 ms (A), and as a function of pulse duration, for a pulse frequency of 130 Hz (B) and 390 Hz (C). In these panels, foundational case one (mean-field coupled stimulation, parameters corresponding to figures 3(B)−(D)) is shown in dark green, and foundational case two (direct stimulation coupling, parameters corresponding to figures 4(B)−(D)) is shown in light green. Solid lines correspond to pulsatile waveforms obtained by matching the cumulative absolute intensity of the optimal smooth waveforms, while dashed lines correspond to pulsatile waveforms obtained by matching the energy of the optimal smooth waveforms. Dotted lines (behind the solid lines where they are not visible) correspond to the smooth optimal waveforms. Panels (D)−(I) show the smooth optimal waveform in red and the pulsatile approximation in black denoted by *u*_*p*_(*t*) (top), as well as the resulting activity of the fast population (bottom). Pulse frequencies/durations are as follow: 135 Hz/0.5 ms in (D) and (G), 130 Hz/4.8 ms in (E) and (H), 390 Hz/2.0 ms in (F) and (I).

Optimal waveforms parametrised by Fourier coefficients may be applicable to stimulation modalities such as tACS, but are not directly applicable to stimulation modalities that can only generate square pulses (e.g. DBS). However, these smooth optimal waveforms can be approximated using pulsatile waveforms. We suggest different ways of doing so, and compare the resulting pulsatile waveforms to the corresponding optimal smooth waveforms in terms of their effects on PAC in the SL and WC models presented before. We approximate smooth waveforms using regularly spaced square pulses, with a certain pulse frequency and pulse duration. The amplitude (intensity) of each pulse is simply given by the amplitude of the smooth waveform at the center of the pulse (as shown for e.g. figure 14(E1)), with a scaling factor determined to either match the energy of the smooth waveform (i.e. ∫ *u*(*t*)^2^d*t*), or its cumulative absolute intensity (i.e. ∫|*u*(*t*)|d*t*). Model simulation with pulsatile waveforms required to use Euler’s method, as a variable step solver (used in the rest of this work) would lead to pulse durations varying with the integration step.

**Figure 15:**
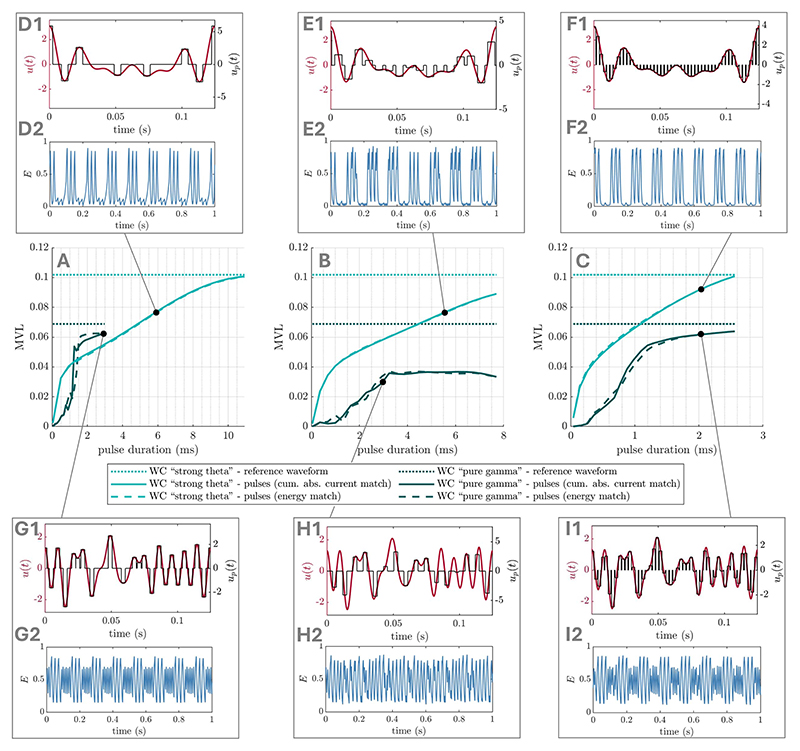
Optimal smooth waveforms can be approximated with pulses—WC model. MVL is shown as a function of pulse duration for pulses centered on the local peaks of the smooth waveform (A), as well as for a pulse frequency of 130 Hz (B) and 390 Hz (C). In these panels, the strong theta case (parameters corresponding to figures 7(B)−(D)) is shown in light green, and the pure gamma case (parameters corresponding to figures 8(B)−(D)) is shown in dark green. Solid lines correspond to pulsatile waveforms obtained by matching the cumulative absolute intensity of the optimal smooth waveforms, while dashed lines correspond to pulsatile waveforms obtained by matching the energy of the optimal smooth waveforms. Dotted lines correspond to the smooth optimal waveforms. Panels (D)−(I) show the smooth optimal waveform in red and the pulsatile approximation in black (top), as well as the resulting activity of the fast population (bottom). Pulse frequencies/durations are as follow: 75.4 Hz(average)/5.9 ms in (D), 130 Hz/5.8 ms in (E) and (I), 390 Hz/2.0 ms in (F), 197 Hz(average)/3.0 ms in (G), and 130 Hz/2.9 ms in (H).

For the SL models investigated in foundational cases one (section 2.1.1) and two (section 2.1.2), relatively low pulse frequencies (135 Hz for 0.5 ms pulse duration) are sufficient for the corresponding pulsatile waveform to increase PAC as much as the smooth optimal waveform when matching cumulative absolute intensity (figure 14(A)). When matching waveform energy, pulse frequency has to be markedly increased to notably affect PAC (figure 14(A)). Alternatively, pulse duration can be lengthened while maintaining a low pulse frequency (figures 14(B) and (C)). For the SL models investigated in the general stimulation coupling case (section 2.1.3), higher pulse frequencies are generally required to match the effects on PAC of the smooth optimal waveforms (around 360 Hz for 0.5 ms pulse duration, see figure S.11 in supplementary material). This is also the case with the WC models investigated in section 2.2, see figure S.12 in supplementary material. However, increasing pulse duration can considerably lower the pulse frequency required (figures 15(B) and (C)). Moreover, irregular pulse spacing chosen such that pulses are centered on the local peaks of the smooth waveform (as shown in figures 15(D1) and (G1)) can further reduce the (average) pulse frequency required to 75 Hz (10 ms pulse) for the strong theta case, and to 197 Hz (3 ms pulse) for the pure gamma case

## 3 Discussion

In this work, we developed a framework to guide the development of optimal PAC-enhancing stimulation. Our framework is for stimulation acting on the neural population generating the fast rhythm, and assumes that neither stimulation nor the fast rhythm significantly affect the slow rhythm (assumed to be generated by another neural population). Using a SL model, we showed that the ARC of the fast population determines which Fourier coefficients should be included and optimised in the stimulation waveform.

Specifically, if the amplitude response to stimulation of the fast rhythm does not depend on its phase, optimal stimulation is at the slow frequency *f_s_* (figure 2(A)). If the amplitude response of the fast rhythm does depend on its phase and its mean is negligible, then the optimal stimulation is a combination of the fast frequency *f_f_*, as well as *f_f_ ± f_s_* (and corresponding harmonics if there are strong harmonics in the ARC of the fast population), see figure 2(B). Otherwise, the optimal stimulation is a combination of these two strategies (figure 2(C)). Neighbouring frequency components may be added if the result is not satisfactory, potentially indicating a dependence of the amplitude response on the amplitude of the fast population.

Additionally, the predictions obtained with the SL model appeared to carry over in several dynamical regimes of a more realistic neural mass model representing interacting neural populations, the WC model (figure 9). Moreover, we showed in the SL model that changes in the balance between Fourier amplitudes as a function of the strength of endogenous fast and slow oscillations are relatively minor but can be explained intuitively (figure 11). We also established that when stimulation includes fast frequency component, it is likely that locking these with the fast population (as specified by the ARC) will be necessary. When stimulation does not include fast components, the importance of phase alignment between stimulation and the slow rhythm depends on PAC levels in the absence of stimulation, and on whether overriding the existing phase-amplitude relationship is acceptable (figure 12). Finally, for neuromodulation modalities that can only generate square waves, the optimal waveforms predicted by our framework can be approximated by pulsatile waveforms.

### 3.1 Modelling PAC generation

Models with various levels of biophysical details have been used to investigate PAC. For example, detailed single and multi-compartment models can generate PAC (see [46] for a review), and the emergence of PAC was investigated in a simulated hippocampal CA1 microcircuit with morphologically detailed neurons [47]. Neural mass models with various types of coupling can also produce PAC, from simple *E* − *I* loops [44] to realistic circuits comprising four cortical layers and dozens of populations [48]. The canonical types of population interactions leading to PAC have been reviewed in [49] (our models correspond to unidirectional coupling from a slow population to a fast population), and the bifurcation types responsible for PAC in these models are studied in [50]. Importantly, the effects of brain stimulation on PAC were only explored in a couple of modelling studies to date, namely in a neuronal network consisting of one thousand cells simulated in NEURON [51], and recently in a model connecting a biophysically-detailed representation of the hippocampus with Kuramoto oscillators portraying input from the medial septum [52].

We chose the SL model for its ability to represent a neural oscillator with a phase and an amplitude variable going through a Hopf bifurcation, and for its analytical tractability which allowed us to gain insights into optimal PAC-enhancing stimulation. We chose the WC model to test the predictions obtained with the SL model since the WC model has been proposed as a canonical E-I circuit to generate PAC [44] and has been commonly used to study neural oscillations and optimise therapeutic brain stimulation [44, 53–60]. Crucially, the WC model is a relatively inexpensive to simulate neural mass model (as opposed to models requiring to simulate individual neurons), which makes numerical optimisation of the stimulation waveform possible. Additionally, in both our SL and WC models, the fast rhythm can be periodically inhibited by the slow rhythm as in detailed neuron models reviewed in [46], but the fast population can also be quiescent (*δ* ⩽ 0 in the SL model and trough of the strong theta regime in the WC model). In that case, fast oscillations are only brought about by the rising slow input causing the model to traverse a Hopf bifurcation [27, 44] (see the WC model strong theta case in section 2.2).

### 3.2 Comparing predictions with experimental data

Results of recent experimental studies are in line with some of the predictions made in this work. In particular, bursts of stimulation phased-locked to the peak of the slow rhythm were found to increase PAC compared to baseline and to stimulation provided at the trough of the slow rhythm [25, 26]. These could correspond to purely excitatory pulses acting through the mechanism presented in section 2.1.1 (see figure 2(A)), or to pulses with excitatory and inhibitory components acting through the mechanism presented in section 2.1.2 (see figure 2(B)) where phase-alignment with the peak of the fast oscillations happens through entrainment. Other studies reported improvements in memory performance [61], motor skill acquisition [62], and cognitive task performance [63] after open-loop transcranial alternative current stimulation with bursts of gamma stimulation superimposed to the peak of a theta stimulation waveform. No improvement was reported in [61, 62] when gamma bursts are superimposed to the trough of the theta stimulation waveform. Although PAC was not measured during stimulation, these behavioural improvements are likely mediated by an increase in PAC. The effective stimulation waveform in these studies correspond to the combination of mechanisms one and two, and is similar to the optimal waveform in the WC model strong theta case (see figure 9(A)). Since stimulation was open-loop, entrainment of both the slow and fast rhythms may have played a role. Furthermore, the additive effect of both mechanisms (i.e slow and fast components of the stimulation waveform) on memory performance was confirmed in [61], as well as the frequency specificity of the fast component of the stimulation waveform.

Nevertheless, more experimental work is required to validate our framework, in particular with regards to the relationship between the characteristics of the ARC of the fast population and the optimal PAC-enhancing waveform. The ARC of the fast population of interest could be measured experimentally using phase-locked stimulation as in previous studies [35, 45]. Recent advances in continuous real-time phase estimation with zero filter delay [64] (also see link to code in [35]) make phase-locking to fast oscillations feasible (robust phase-locking was achieved at 40 Hz in [35]). Another method was recently shown to reliably estimate in real-time the phase of oscillations up to 250 Hz in synthetic data [65]. These advances will also be key to phase-locking stimulation to the fast rhythm according to the mechanism described in figure 2(B).

### 3.3 Limitations

In this work, the slow rhythm involved in PAC was considered to be generated by an external neural population (for example by the medial septum in the case of hippocampal theta-gamma PAC). It was assumed that neither the fast population nor stimulation significantly influence the slow rhythm. This is justified if stimulation is local to the fast population, and the influence of the fast population on the slow rhythm averages out on the slow timescale [27] or the projections from the fast population to the slow population are weak. However, if stimulation significantly affects the slow rhythm, the impact on PAC can be substantial as shown recently [52]. Including in our framework the potential effects of stimulation on the slow rhythm will be a focus for future work. Additionally, how our framework may generalise to more detailed models of neural populations has not been studied beyond the WC. Since the SL model used to develop the framework is the normal form of a Hopf bifurcation, we can speculate that our predictions may hold in more detailed models (e.g. including detailed representations of individual neurons) that operate in the vicinity of a Hopf bifurcation.

Further limitations of this study include the absence of noise and the absence of synaptic plasticity in our models, as well as technical assumptions in derivations (the ARC is assumed to be a separable function of phase and amplitude, and we assumed small stimulation and *ω_f_* ≫ *ω_s_* or entrainment). We also used a non-normalised MVL measure, although this is offset by the fact that stimulation waveform energy is constrained in numerical optimisations. Numerical optimisations were limited by available supercomputing resources, and the optimal balance of stimulation waveform Fourier coefficients across model parameters could not be investigated with a finer Fourier amplitude grid in the SL model, or at all in the WC model. Lastly, our framework assumes a smooth stimulation waveform (as in tACS). While we propose different ways of approximating the smooth optimal waveforms with pulsatile waveforms for neuromodulation devices that can only generate pulses (section 2.4), the pulse frequencies and durations required may not be achievable by some of these devices.

### 3.4 Conclusion

We have presented a framework to design optimal PAC-enhancing (or PAC-decreasing) stimulation based on the amplitude response of the fast population, assuming that stimulation acts solely on the neural population generating the fast rhythm. We hope that this framework can help guide the development of innovative therapeutic brain stimulation aiming at restoring healthy levels of PAC, for example in patients with AD, where theta-gamma PAC is abnormally low and correlates with cognitive symptoms.

## Supplementary Material

Supplementary material

Appendix

## Data Availability

No new data were created or analysed in this study.
