# Supplementary material for: How to design optimal brain stimulation to modulate phase-amplitude coupling?
Source: J Neural Eng. Author manuscript; Available in PMC 2024 Aug 1. (PMC7616267; doi:10.1088/1741-2552/ad5b1a)
Supplement: Appendix [file EMS197477-supplement-Appendix.pdf]

y
d
l,
k
e

Appendix

In this section, we provide methodological details pertaining to PAC measurement, numerical optimisation of the stimulation waveform to enhance PAC, the perturbation analysis of stimulation Fourier coefficients, the WC model, and the estimation of ARCs in the WC model. We also present derivation details for the analytical approaches pursued in the SL model with direct stimulation coupling and general stimulation coupling.

A.1. Measuring PAC levels in simulations

In our simulations, the MVL is obtained in discrete time as

$$\mathrm{MVL} = \frac{1}{N_t}\left|\sum_{j=1}^{N_t} \rho_f\left(t_j\right) \mathrm{e}^{\mathrm{i}\omega_s t_j}\right|, \tag{12}$$

where N_t is the number of sampling points in the time period considered. The amplitude ρ_f is the Hilbert amplitude of the filtered model output (model output taken as $\Re(z_f)$ for SL [42], and as E for WC). A third-order butterworth filter with zero-phase filtering is used to avoid phase distortions, and the Hilbert amplitude is given by the modulus of the analytic signal constructed with the Hilbert transform. It was also found empirically that stimulation waveform optimisations become unstable for narrow filter half-widths such as $\Delta f_f = 5$ Hz. We therefore use filter half-widths of 10 or 20 Hz (see corresponding figure captions for specific values). Note that the phase of the slow signal is given in equation (12) by $\omega_s t_i$ since in the examples considered, the slow signal is always $\cos(\omega_s t_i)$, or a scaled and shifted version of it.

A.2. Numerical optimisation of stimulation waveform

To test our theory in the SL and WC models, we numerically optimise the Fourier coefficients of the stimulation waveform to maximise PAC. Each optimisation on a given variant of the SL or WC model consists of many local optimisations starting from different initial values of the stimulation Fourier coefficients. These are drawn from a uniform distribution on a logarithmic scale between 10^{-3} and 1, and then rescaled so that the energy of each initial waveform is the target stimulation energy Ξ. Local optimisations are performed in Matlab using the non-linear optimiser fmincon based on the interior-point algorithm [66], under the constraint that the energy of the waveform (given by $\sum_j^{N_u}[a_j^2 + b_j^2]/2$) is kept within a small tolerance ($\epsilon = 0.1$) of the target waveform energy Ξ. The target energy is chosen for each model variant so that the optimal stimulation has a significant effect on PAC (see corresponding figure captions for the specific target values). Hard bounds between $\pm\sqrt{2\Xi}$ are also enforced during optimisation. For optimisation speed and accuracy, the models are simulated using Matlab's solver ode113 (variable-step, variable-order Adams-Bashforth-Moulton solver of orders 1–13). At each optimisation step, models are simulated for 30 s for variants of the SL model, and 20 s for variants of the WC model (reduced duration to improve optimisation speed). The transient is discarded by removing the first third of the simulation output.

The objective function to minimise during optimisation is

$$\mathrm{cost} = -\mathrm{MVL} + \frac{1}{\mu\left(2N_u^{\mathrm{opt}}\right)\sqrt{2\Xi}}\|\mathbf{x}\|_1, \tag{13}$$

where the first term ensures that the level of PAC is maximised (MVL obtained as described in section A.1), and the second term is a regularisation term. The Fourier coefficients being optimised are denoted $\mathbf{x} = [a_1, \ldots, a_{N_u^{\mathrm{opt}}}, b_1, \ldots b_{N_u^{\mathrm{opt}}}]$, and the norm $\|\mathbf{x}\|_1 = \sum_i |x_i|$ denotes the 1-norm. The regularisation term is scaled using the absolute energy bound for Fourier coefficients during the optimisation ($\sqrt{2\Xi}$), the number of Fourier coefficients being optimised ($2N_u^{\mathrm{opt}}$), and a regularisation parameter μ. Regularisation was only used to guide the more challenging optimisations—we used $\mu = 1$ for the variant of the SL model with dependence on ρ, $\mu = 15$ for the WC models, and a very large number otherwise (no regularisation).

The number of local optimisations was increased when optimising all Fourier coefficients compared to when optimising only the Fourier coefficients predicted by theory. At least 3000 local optimisations were performed in the latter case, while at least 11 000 local optimisations were performed in the former case. This was to mitigate the increase in the number of optimised parameters (within the limits of supercomputing resources available). In both cases, the best-ranked parameters coming out of the local optimisations were put through one other round of local optimisation (except for the SL models with mean-field and direct stimulation coupling, which were easier to optimise).

A.3. Perturbation analysis

As an additional investigation into which Fourier coefficients of the stimulation waveform are key to enhancing PAC, we perturb individual Fourier coefficients and assess changes in MVL. For each of the model variants considered, we perturb the n_{pert}-best PAC-enhancing waveforms (obtained from numerically optimising all the Fourier coefficients), as well as n_{pert} random waveforms. Random waveforms are generated by drawing Fourier coefficients from a uniform distribution and re-scaling the coefficients such that the waveform energy is Ξ. We take $n_{\mathrm{pert}} = 100$ for SL models, and $n_{\mathrm{pert}} = 200$ for WC models (more variability in the latter case). We perturb each Fourier coefficient in turn by adding the perturbation $\sqrt{\Xi}/10$, where Ξ is the waveform energy before perturbation. We measure the absolute change in PAC as $|\mathrm{MVL} - \mathrm{MVL}_0|$, where MVL is the PAC level with the perturbation, and MVL_0 the PAC level in the absence of perturbation. For each of the model variants considered, the absolute change in PAC is averaged separately across the n_{pert}-best PAC-enhancing waveforms and the n_{pert} random waveforms.

We also follow the approach above to show that φ_u does not introduce significant dependences on other Fourier coefficients than those predicted by theory in the SL model. The only difference is that we fit a straight line to the time evolution of θ_f (first third of the data discarded to remove transient), and measure φ_u as its intercept. For each perturbation, we measure the absolute change in φ_u as $|\varphi_u - \varphi_{u_0}|$, where φ_{u_0} is the phase shift in the absence of perturbation. We average absolute differences as in the MVL case above.

A.4. Derivation details for foundational case two

In this section, we derive a relationship between stimulation waveform Fourier coefficients and the amplitude of the fast population in the case of direct coupling in the SL model (foundational case two). This will allow us to gain insights into which Fourier coefficients can have a significant impact on PAC. Using the approximation for θ_f mentioned in section 2.1.2 in the Results, the time evolution of ρ_f is given by equation (10). In the steady-state, solutions with PAC will be periodic with period $2\pi/\omega_s$. Such solutions can therefore be approximated as Fourier series $\rho_f = \sum_{n=-N_\rho}^{N_\rho} c_n e^{n i \omega_s t}$ truncated at order N_ρ. We also have $\rho_f^3 = \sum_{n=-3N_\rho}^{3N_\rho} \Pi_n e^{n i \omega_s t}$, where the Fourier coefficients Π_n can be obtained as functions of the coefficients of ρ. Equation (10) becomes

$$\begin{aligned}
&\sum_{n=-N_\rho}^{N_\rho} n i \omega_s c_n e^{n i \omega_s t} \\
&= - \sum_{n=-3N_\rho}^{3N_\rho} \Pi_n e^{n i \omega_s t} + \sum_{n=-N_\rho}^{N_\rho} \delta c_n e^{n i \omega_s t} \\
&\quad + \sum_{n=-N_\rho}^{N_\rho} \frac{k_s}{2} c_n e^{(n+1) i \omega_s t} + \sum_{n=-N_\rho}^{N_\rho} \frac{k_s}{2} c_n e^{(n-1) i \omega_s t} \\
&\quad + \sum_{\substack{n=-N_u \\ n\neq 0}}^{N_u} \frac{u_n}{2} e^{(n+r) i \omega_s t} e^{i\varphi_u} + \sum_{\substack{n=-N_u \\ n\neq 0}}^{N_u} \frac{u_n}{2} e^{(n-r) i \omega_s t} e^{-i\varphi_u}.
\end{aligned}$$

By manipulating indices and identifying terms corresponding to $e^{n i \omega_s t}$, we obtain

$$\begin{aligned}
0 &= (\delta - n i \omega_s) c_n + \frac{k_s}{2} c_{n-1} + \frac{k_s}{2} c_{n+1} - \Pi_n \\
&\quad + \frac{1}{2}\left(u_{n-r} e^{i\varphi_u} + u_{n+r} e^{-i\varphi_u}\right),
\end{aligned} \tag{14}$$

with $u_0 = 0$, $u_n = 0$ for $|n| > N_u$, $c_n = 0$ for $|n| > N_\rho$, and $\Pi_n = 0$ for $|n| > 3N_\rho$.

Most of the PAC strength is captured by the first harmonic of ρ, we therefore consider equation (14) for $c_0 = \rho_0$, $c_1 = \rho_1 e^{i\theta_1}$ $(c_{-1} = \bar{c_1})$, and $c_n = 0$ for $|n| > 1$. Since $\Pi_0 = 6c_0c_1c_{-1} + c_0^3$, and $\Pi_1 = 3c_1c_0^2 + 3c_{-1}c_1^2$, we get

$$\begin{aligned}
n = 0: \quad 0 &= \delta c_0 + \frac{k_s}{2} c_{-1} + \frac{k_s}{2} c_1 - 6c_0c_1c_{-1} - c_0^3 \\
&\quad + \frac{1}{2}\left(u_{-r} e^{i\varphi_u} + u_r e^{-i\varphi_u}\right), \\
n = 1: \quad 0 &= (\delta - i\omega_s) c_1 + \frac{k_s}{2} c_0 - 3c_1c_0^2 - 3c_{-1}c_1^2 \\
&\quad + \frac{1}{2}\left(u_{1-r} e^{i\varphi_u} + u_{1+r} e^{-i\varphi_u}\right).
\end{aligned}$$

These two equations translate to three equations in ρ_0, ρ_1, and θ_1 given by

$$0 = \delta\rho_0 + \frac{k_s}{2}\rho_1\cos\theta_1 - 6\rho_0\rho_1^2 - \rho_0^3 + \Re\left(u_r e^{-i\varphi_u}\right), \quad (15)$$

$$0 = \delta\rho_1\cos\theta_1 + \omega_s\rho_1\sin\theta_1 + \frac{k_s}{2}\rho_0 - 3\rho_1\rho_0^2\cos\theta_1 - 3\rho_1^3\cos\theta_1 + \frac{1}{2}\Re\left(\bar{u}_{r-1}e^{i\varphi_u} + u_{1+r}e^{-i\varphi_u}\right), \quad (16)$$

$$0 = \delta\rho_1\sin\theta_1 - \omega_s\rho_1\cos\theta_1 - 3\rho_1\rho_0^2\sin\theta_1 - 3\rho_1^3\sin\theta_1 + \frac{1}{2}\Im\left(\bar{u}_{r-1}e^{i\varphi_u} + u_{1+r}e^{-i\varphi_u}\right). \quad (17)$$

We demonstrate numerically through a perturbation approach that the phase shift φ_u (which also depends on the stimulation waveform) does not introduce dependences on additional Fourier coefficients than those explicitly present in these equations (see figure S.1(A) in supplementary material and methodological details in section A.3, perturbation of size $\sqrt{\Xi}/10$). We present the insights obtained from these equations and test predictions arising from them in section 2.1.2 in the Results.

A.5. Derivation details for general stimulation coupling in the SL model

We generalise the derivation presented in the previous section to a general stimulation coupling, where the ARC of the fast population is a separable function of θ_f and ρ_f (see section 2.1.3 in the Results), with a view to gaining insights into which Fourier coefficients can have a significant impact on PAC. From equation (1) with general coupling, the time evolutions of ρ_f and θ_f are given by

$$\dot{\rho}_f = -\rho_f^3 + [\delta + k_s\cos(\omega_s t)]\rho_f + g(\rho_f)\sum_{n=-N_a}^{N_a}\alpha_n e^{ni\theta_f}u(t),$$
$$\dot{\theta}_f = \omega_f + \mathrm{PRC}(\theta_f,\rho_f)\,u(t). \quad (18)$$

As previously, equation (18) can be approximated by

$$\dot{\rho}_f = -\rho_f^3 + [\delta + k_s\cos(\omega_s t)]\rho_f + g(\rho_f) \times \sum_{n=-N_a}^{N_a}\alpha_n e^{ni(r\omega_s t+\varphi_u)}u(t). \quad (19)$$

Since ρ_f is periodic, $g(\rho_f)$ can be approximated by a truncated Fourier series $g(\rho_f) = \sum_{n=-N_\gamma}^{N_\gamma} d_n e^{ni\omega_s t}$. Note that each d_n depends on the Fourier coefficients of ρ_f. Using the Fourier expansions of the various terms as before, equation (19) becomes

$$\sum_{n=-N_\rho}^{N_\rho} ni\omega_s c_n e^{ni\omega_s t} = -\sum_{n=-3N_\rho}^{3N_\rho}\Pi_n e^{ni\omega_s t} + \sum_{n=-N_\rho}^{N_\rho}\delta c_n e^{ni\omega_s t} + \sum_{n=-N_\rho}^{N_\rho}\frac{k_s}{2}c_n e^{(n+1)i\omega_s t} + \sum_{n=-N_\rho}^{N_\rho}\frac{k_s}{2}c_n e^{(n-1)i\omega_s t} + \sum_{k=-N_a}^{N_a}\sum_{m=-N_\gamma}^{N_\gamma}\sum_{\substack{l=-N_u\\ l\neq 0}}^{N_u} d_m\alpha_k u_l e^{(kr+l+m)i\omega_s t}e^{ki\varphi_u}.$$

Using $n = kr + l + m$, we have

$$\sum_{k=-N_a}^{N_a}\sum_{m=-N_\gamma}^{N_\gamma}\sum_{\substack{l=-N_u\\ l\neq 0}}^{N_u} d_m\alpha_k u_l e^{(kr+l+m)i\omega_s t} = \sum_{k=-N_a}^{N_a}\sum_{m=-N_\gamma}^{N_\gamma}\sum_{\substack{n=kr-N_u\\ n\neq kr+m}}^{kr+N_u} d_m\alpha_k u_{n-kr-m}e^{ni\omega_s t}.$$

By manipulating indices and identifying terms corresponding to $e^{ni\omega_s t}$, we obtain

$$0 = (\delta - ni\omega_s)c_n + \frac{k_s}{2}c_{n-1} + \frac{k_s}{2}c_{n+1} - \Pi_n + \sum_{k=-N_a}^{N_a}\sum_{m=-N_\gamma}^{N_\gamma} d_m\alpha_k u_{n-kr-m}e^{ki\varphi_u}, \quad (20)$$

with $u_0 = 0$, $u_n = 0$ for $|n| > N_u$, $c_n = 0$ for $|n| > N_\rho$, $\Pi_n = 0$ for $|n| > 3N_\rho$, $a_n = 0$ for $|n| > N_a$, and $d_n = 0$ for $|n| > N_\gamma$.

As before, most of the PAC strength is captured by the first harmonic of ρ with coefficients $c_0 = \rho_0$, $c_1 = \rho_1 e^{i\theta_1}$ $(c_{-1} = \bar{c}_1)$. Neglecting the higher order harmonics of ρ, the coefficients d_m will only depend on c_0 and c_1. We have

$$n = 0:\quad 0 = \delta c_0 + \frac{k_s}{2}c_{-1} + \frac{k_s}{2}c_1 - 6c_0c_1c_{-1} - c_0^3 + \sum_{k=-N_a}^{N_a}\sum_{m=-N_\gamma}^{N_\gamma} d_m(c_0,c_1)\,\alpha_k u_{-kr-m}e^{ki\varphi_u},$$

$$n = 1:\quad 0 = (\delta - i\omega_s)c_1 + \frac{k_s}{2}c_0 - 3c_1c_0^2 - 3c_{-1}c_1^2 + \sum_{k=-N_a}^{N_a}\sum_{m=-N_\gamma}^{N_\gamma} d_m(c_0,c_1)\,\alpha_k u_{1-kr-m}e^{ki\varphi_u}.$$

These two equations translate to three equations in ρ_0, ρ_1, and θ_1 given by

$$0=\delta\rho_0+\frac{k_s}{2}\rho_1\cos\theta_1-6\rho_0\rho_1^2-\rho_0^3+2\Re\left(\bar{d}_1(\rho_0,\rho_1,\theta_1)\,\alpha_0u_1\right)$$
$$+2\sum_{k=-N_a}^{N_a}\sum_{m=-N_\gamma}^{N_\gamma}\Re\left(d_m(\rho_0,\rho_1,\theta_1)\,\overline{\alpha}_k u_{kr-m}\mathrm{e}^{-ki\varphi_u}\right)$$
$$+2\sum_{m=1}^{N_\gamma}\Re\left(\bar{d}_m(\rho_0,\rho_1,\theta_1)\,\alpha_0u_m\right), \tag{21}$$

$$0=\delta\rho_1\cos\theta_1+\omega_s\rho_1\sin\theta_1+\frac{k_s}{2}\rho_0-3\rho_1\rho_0^2\cos\theta_1-3\rho_1^3\cos\theta_1$$
$$+\sum_{k=-N_a}^{N_a}\sum_{m=-N_\gamma}^{N_\gamma}\Re\left(d_m(\rho_0,\rho_1,\theta_1)\,\alpha_k\bar{u}_{kr+m-1}\mathrm{e}^{ki\varphi_u}\right), \tag{22}$$

$$0=\delta\rho_1\sin\theta_1-\omega_s\rho_1\cos\theta_1-3\rho_1\rho_0^2\sin\theta_1-3\rho_1^3\sin\theta_1$$
$$+\sum_{k=-N_a}^{N_a}\sum_{m=-N_\gamma}^{N_\gamma}\Im\left(d_m(\rho_0,\rho_1,\theta_1)\,\alpha_k\bar{u}_{kr+m-1}\mathrm{e}^{ki\varphi_u}\right). \tag{23}$$

As before, we demonstrate numerically through a perturbation approach detailed in section A.3 that the phase shift φ_u (which also depends on the stimulation waveform) does not introduce dependences on additional Fourier coefficients than those explicitly present in these equations (see figure S.1(C) for $g(\rho_f)=1$, and figure S.1(D) for $g(\rho_f)=1/\rho_f$ in supplementary material). We present the insights obtained from these equations and test predictions arising from them for two examples of $g(\rho_f)$ in section 2.1.3 in the Results.

A.6. Wilson–Cowan model

To test whether the predictions obtained from the SL model may apply in a more biologically realistic context, we make use of a neural mass model, the Wilson–Cowan model. The WC model depicts the interactions of a population of excitatory neurons, whose activity is denoted by E, and a population of inhibitory neurons, whose activity is denoted by I (see figure 6). Two heuristically derived mean-field equations [43] describe the time evolution of the populations' activities as

$$\begin{cases}\tau\dot{E}=-E+f(\eta_E+w_{EE}E-w_{IE}I+u(t))\\ \tau\dot{I}=-I+f(\eta_I+w_{EI}E),\end{cases} \tag{24}$$

with w_{PR} the weight of the projection from population 'P' to population 'R', η_P the external input to population 'P', $u(t)$ the external stimulation, and τ a time constant (assumed to be the same for both populations). As in [44], the function f is the sigmoid function

$$f(x)=\frac{1}{1+\mathrm{e}^{-\beta(x-1)}},$$

parametrised by a steepness parameter β. To get PAC in the absence of stimulation, we follow [44] and provide the slow input

$$\eta_E=c_1\cos(\omega_s t)+c_2$$

to the excitatory population (c_1 is set to zero in the pure gamma case). We consider two examples with model parameters leading to dynamically distinct behaviours (strong theta case and pure gamma case, see section 2.2). The parameters used in the simulations are reported in table 1.

A.7. Obtaining amplitude-response curves in the Wilson–Cowan model

Assessing whether predictions made with the SL model may hold for the WC model requires the ARC of the WC model in the examples considered. Thus, we approximate the ARC of the excitatory population (the population receiving stimulation) in the strong theta and pure gamma cases as follows. Our approach is inspired by [67], and does not rely on the more complicated definitions of the amplitude response involving isostables [59, 68–71]. The intuition behind our approach is as follows. The instantaneous change in the system's state due to stimulation will in general change both the phase and the amplitude of the system. In the two-dimensional (E,I) phase space, the instantaneous change in phase due to a small stimulation at a given point on a trajectory can be obtained from the component of the shift due to stimulation that is tangent to the trajectory at the stimulation point. Conversely, the instantaneous change in amplitude is given by the component of the shift due to stimulation that is normal to the trajectory at the stimulation point. To obtain the ARC for a periodic trajectory of interest, we therefore need to compute the normal component of the change in state due to stimulation at a number of points along the trajectory. These points are chosen such that they span the full range of phases on the periodic trajectory and capture the phase dependence of the ARC with sufficient detail (for the amplitude of the periodic orbit considered).

Table 1. Parameters of the Wilson–Cowan model used in simulations. The strong theta case correspond to figure 7, and the pure gamma case to figure 8. Parameters of the strong theta case are taken from [44].

Model	w_{EE}	w_{IE}	w_{EI}	τ	β	c_1	c_2	ω_s	η_I
strong theta case	2.4	2	2	0.0032	4	0.05	0.385	$2\pi\times 8$	0
pure gamma case	4.2676	9.2272	1.1640	0.0054	4.1819	0	2.4646	NA	0.3242

Along the periodic trajectories of interest, we therefore calculate at regular time intervals the instantaneous change in the activity of the E population due to stimulation ΔE_u. As per equation (24), during a time step Δt, the instantaneous change in E due to both stimulation and the dynamics of the system is given by

$$\Delta E_{u+\mathrm{dyn}}(E,I) = \frac{\Delta t}{\tau}\left[-E + f(\eta_E + w_{EE}E - w_{IE}I + u(t))\right],$$

while the instantaneous change in E due to the dynamics alone is given by

$$\Delta E_{\mathrm{dyn}}(E,I) = \frac{\Delta t}{\tau}\left[-E + f(\eta_E + w_{EE}E - w_{IE}I)\right].$$

Thus, we obtain the instantaneous change in the activity of the E population due to stimulation as

$$\begin{aligned}\Delta E_u(E,I) &= \Delta E_{u+\mathrm{dyn}} - \Delta E_{\mathrm{dyn}},\\ \Delta E_u(E,I) &= \frac{\Delta t}{\tau}\left[f(\eta_E + w_{EE}E - w_{IE}I + u)\right.\\ &\quad\left. - f(\eta_E + w_{EE}E - w_{IE}I)\right],\end{aligned}$$

where we choose $u = 0.3$ in our numerical estimations of the ARC. Since stimulation is only provided to the excitatory population (see equation (24)), the instantaneous change in the activity of the I population due to stimulation is $\Delta I_u = 0$. At each point considered along the trajectory, we also get the tangent vector to the trajectory as a numerical approximation of $\begin{bmatrix}\dot{E}\\ \dot{I}\end{bmatrix}$ using central differences. We then obtain $\mathbf{n}$ as the counter-clockwise unit normal vector to the tangent vector. Finally, we approximate the ARC as the projection of the change in population activity due to stimulation onto the normal vector at each point considered along the trajectory of interest,

$$\mathrm{ARC} = -\begin{bmatrix}\Delta E_u\\ \Delta I_u\end{bmatrix}\cdot\mathbf{n},$$

where the negative sign gives a positive value for an increase in amplitude. Each point along the trajectory where the change in amplitude was computed is assigned a phase given by $\theta_f = \omega_f t$, which allows us to re-parametrise the ARC as a function of phase. This ARC approximation process is illustrated in figure S.6 in the supplementary material. In the strong theta case, the trajectory considered is the on-stimulation gamma cycle (see figure S.6(A)). In the pure gamma case, the significant changes in dynamics for low and high amplitudes require to consider both the low-amplitude trajectory on stimulation (figure S.6(B1)), and the high-amplitude periodic trajectory (similar on and off stimulation, taken off stimulation in figure S.6(B2)).
